# Novel Charge-Switch Derivatization Method Using 3-(Chlorosulfonyl)benzoic Acid for Sensitive RP-UHPLC/MS/MS Analysis of Acylglycerols, Sterols, and Prenols

**DOI:** 10.1021/acs.analchem.4c06496

**Published:** 2025-03-28

**Authors:** Ondřej Peterka, Yasmin Kadyrbekova, Robert Jirásko, Zuzana Lásko, Bohuslav Melichar, Michal Holčapek

**Affiliations:** † University of Pardubice, Faculty of Chemical Technology, Department of Analytical Chemistry, Studentská 573, 532 10 Pardubice, Czech Republic; ‡ Palacký University Medical School and University Hospital Olomouc, Faculty of Medicine and Dentistry, Department of Oncology, I.P. Pavlova 6, 775 20 Olomouc, Czech Republic

## Abstract

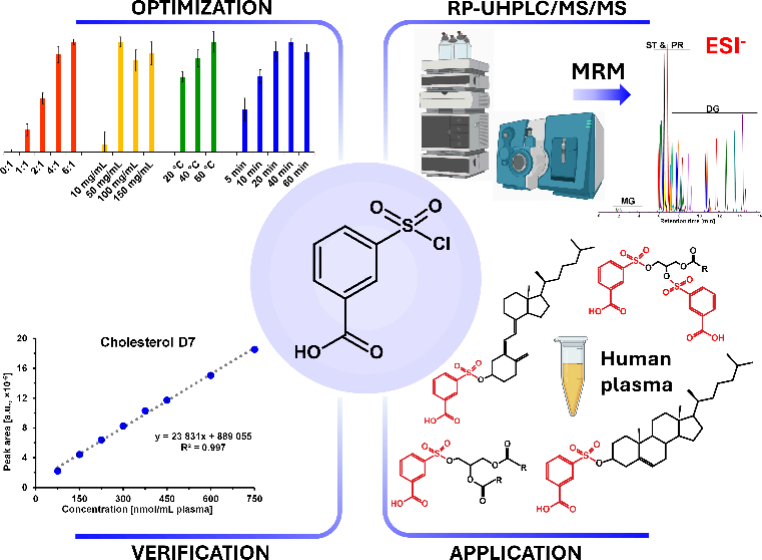

Chemical derivatization involves the reaction of an analyte with a derivatization agent to modify its structure, improving the peak shape, chromatographic performance, structural analysis, ionization efficiency, and sensitivity. A novel derivatization method using 3-(chlorosulfonyl)benzoic acid is developed for the determination of monoacylglycerols, diacylglycerols, free sterols, and tocopherols using the reversed-phase ultra-high-performance liquid chromatography–tandem mass spectrometry (RP-UHPLC/MS/MS) method in the negative ion mode. The chromatographic and mass spectrometric properties of derivatized lipids are investigated by using 29 lipid standards spanning four lipid classes. The derivatization process is optimized using pooled plasma spiked by 9 internal standards, achieving an optimal yield with a reaction time of 40 min at 60 °C. The stability of the derivatives is confirmed, with short-term stability maintained for 10 h at 4 °C and long-term stability preserved for 5 days at −80 °C. The repeatability and reproducibility are verified by one/two operator(s), which underscores the simplicity and robustness of the method, and calibration curves with high linear regression coefficients illustrate the accuracy of the method. The derivatization approach, which combines RP-UHPLC/MS/MS and the use of specific fragmentation patterns, significantly reduces limits of detection, reaching 15–25 pmol/mL for free sterols in plasma. The optimized method is applied to the analysis of human plasma, leading to the identification of 92 lipid species in the targeted lipid classes. This represents a substantial improvement in sensitivity and detection capabilities compared to those of previously reported methods.

## Introduction

Lipids are essential components of all living organisms and play critical roles in cellular processes, such as energy storage, signaling, and formation of membrane structure.^1^ Lipidomics is the study of the structure, function, and metabolism of lipids. The dysregulation of lipids has been linked to many diseases, such as hypercholesterolemia,^2,3^ Alzheimer’s disease,^4^ cardiovascular diseases,^5^ and cancer.^6^ Biomarker research is mostly focused on polar lipids, and many potential biomarkers have been published so far.^7^ However, nonpolar lipids, especially monoacylglycerols, diacylglycerols, and sterols, are precursors of signaling lipids and hormones, which play an essential role in the human metabolism.^8^ Unfortunately, their analysis is complicated, and therefore, these molecules are often neglected.

Mass spectrometry (MS) coupled with separation techniques, especially with liquid chromatography (LC)^9^ or supercritical fluid chromatography (SFC),^10^ is the main approach in the lipidomic analysis. However, this approach can be divided into a lipid class separation approach represented by hydrophilic interaction chromatography (HILIC) or normal-phase chromatography and a lipid species separation approach represented by reversed-phase (RP) chromatography. The lipid class separation approach is based on the separation of lipids according to the polar headgroup, resulting in the coelution of all endogenous lipid species from the same lipid class in one chromatographic peak, which does not allow identification and quantitation of individual isomers. On the other hand, the lipid species separation approach separates lipids based on the length of fatty acyl chains and the number of double bonds (DB), allowing the separation of isomers, which leads to a higher level of structural information.^11,12^

The analysis of neutral lipids in biological samples remains a challenge, from sample preparation to identification and quantification. Low ionization efficiency, high in-source fragmentation, formation of various adducts, and missing selective multiple reaction monitoring (MRM) transitions limit their analysis,^13^ but the chemical derivatization can significantly improve sensitivity and selectivity of analysis. There are several methods using some derivatization agent reacting with the hydroxyl group(s) of DG and MG, followed by LC/MS analysis, such as benzoyl chloride,^14^*N*-chlorobetainyl chloride,^15^ 2,4-difluorophenyl isocyanate,^16^*N*,*N*-dimethylglycine and *N*,*N*-dimethylalanine,^17^ 1-(1-naphtyl)-ethyl-isocyanate,^18,19^ and 3-nitrophenylboronic acid.^20^ Otherwise, two derivatization approaches are used for free sterols: the direct reaction of the hydroxyl group with a derivatization agent, for example, acetyl chloride,^21^ benzoyl chloride,^14^ dansyl chloride,^22^ tris(2,4,6,-trimethoxyphenyl)phosphonium acetic acid,^23^ sulfur trioxide pyridine,^24^ 4-(dimethylamino)phenyl isocyanate,^25^ picolinic acid,^26^ and dimethylglycine,^27^ or oxidation of the hydroxyl group to ketone, followed by a reaction with a hydrazine-based agent, such as Girard P^28^ and Girard T^29^ reagents. The derivatization can also be used for the determination of a higher level of lipid structures, such as the position of DB using photochemical reactions,^30^ ozonolysis,^31^ or specific derivatization agents.^32,33^

The goal of this work is the development and optimization of new chemical derivatization focused on selected neutral lipids that enable the detection of derivatives by MS in the negative ion mode, which should minimize in-source fragmentation, form preferred adduct ions, and fragments for MRM transitions, leading to high selectivity and sensitivity of the analysis. The RP-UHPLC/MS/MS method is used for the detection and analysis of derivatives. The combination of the derivatization method and RP-UHPLC/MS/MS is used for the qualitative analysis of selected lipid classes in human plasma samples.

## Experimental Section

### Chemicals and Standards

Acetonitrile (CH_3_CN), 1-buthanol (BuOH), methanol (MeOH), 2-propanol (i-PrOH), formic acid (all LC/MS gradient grade), and ammonium carbonate (≥30.0% NH_3_ basis) were purchased from Honeywell (Charlotte, North Carolina, US). Ammonium formate (for MS, ≥99.0%), 3-(chlorosulfonyl)benzoic acid (95%) (Cl-SBA), and pyridine (for HPLC, ≥99.9%) were purchased from Sigma-Aldrich (St. Louis, MO, USA) and LiChrosolv chloroform (stabilized with 2-methyl-2-butene) from Merck (Darmstadt, Germany). Deionized water was prepared using a Milli-Q Reference Water Purification System (Molsheim, France). Lipid standards (Table S1) and internal standards (Table S2) were purchased from Avanti Polar Lipids (Alabaster, AL, USA), Nu-Chek Prep (Elysian, MN, USA), or Merck (Darmstadt, Germany). All stock solutions of lipid standards were prepared in MeOH/CHCl_3_ (1:1, v/v) and stored at −80 °C. Deuterated IS are typically delivered in chloroform solution, which can be used directly as a stock solution. Mixture of standards and internal standards were prepared by mixing of aliquots from stock solutions of individual lipid species and diluted by a mixture of MeOH/CHCl_3_ (1:1, v/v) to reach final concentrations.

### Plasma Sample

The pooled plasma sample used for the optimization of the derivatization process and identification was prepared by mixing aliquots of 40 human plasma samples (ages 38–58 years and a body mass index of 20–30). Samples of 20 male and 20 female volunteers were obtained from the Palacký University and University Hospital Olomouc, Czech Republic (Table S3). The study was approved by the institutional ethical committee, and all subjects signed an informed consent. All plasma samples were stored at −80 °C.

### Protein Precipitation

The deproteinization of 10 μL of pooled plasma spiked with 20 μL of an internal standard mixture (IS-Mix) in a 1.5 mL glass vial was performed using 250 μL of BuOH/MeOH (1:1, v/v).^5,34^ The samples were placed in an ultrasonic bath for 10 min at 30 °C. After the samples were cooled to room temperature, an additional 500 μL of BuOH/MeOH (1:1, v/v) was added to minimize losses on the filter. Subsequently, the samples were centrifuged (Hettich EBA 20) at 6000 rpm for 3 min and filtrated with a 0.25 μm cellulose filter (OlimPeak, Teknokroma). The extracts were evaporated under a gentle stream of nitrogen at 35 °C, and the residues were stored at −80 °C for the next experiments or immediately used for the derivatization.

### Derivatization

**Figure 1:**
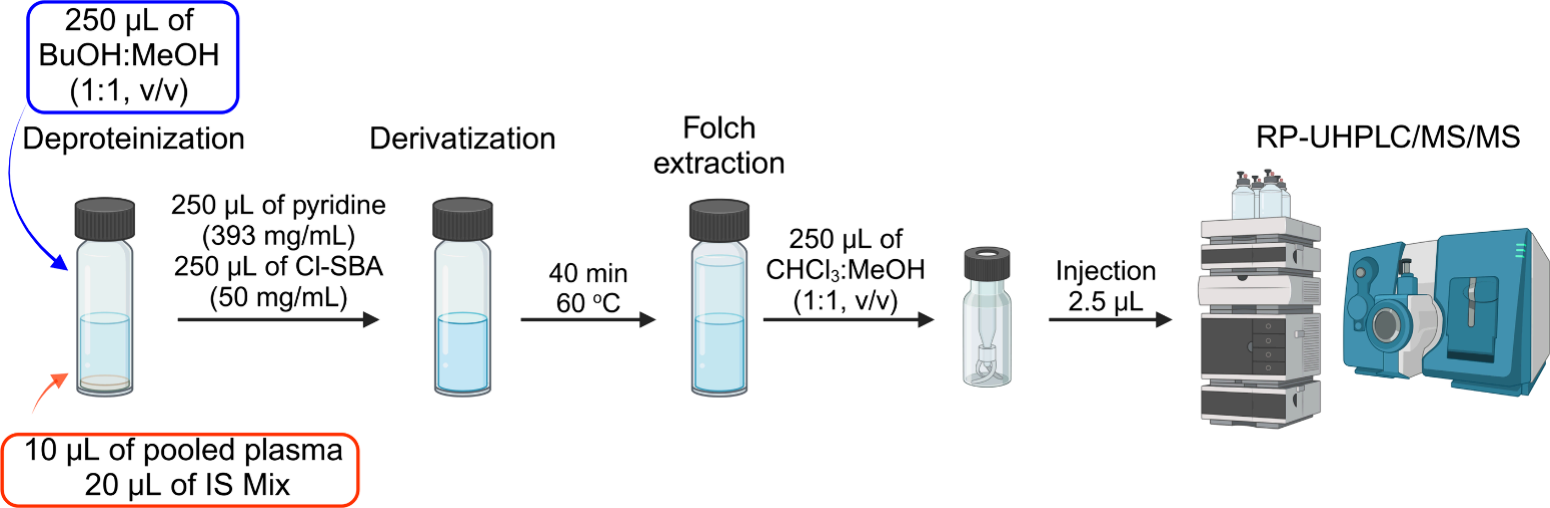
Workflow of the derivatization method for a human plasma sample spiked by the mixture of internal standards measured by the RP-UHPLC/MS/MS method. Figure was created using BioRender.

The sample residue was redissolved in 250 μL of pyridine (392.8 mg/mL in CH_3_CN), and then 250 μL of Cl-SBA (50 mg/mL in CH_3_CN) was added. The reaction mixture was placed in a shaking water bath (150 rpm, Memmert, Schwabach, Germany) at 60 °C for 40 min. Afterward, the reaction was stopped by applying the Folch lipid extraction protocol.^35^ 3 mL of a mixture of CHCl_3_/MeOH (2:1, v/v) and 0.6 mL of water were added to the reaction mixture and stirred (560 rpm, KS 130 shaker, IKA, Staufen, Germany) for 5 min at room temperature. The samples were centrifuged (Hettich EBA 20) at 6000 rpm for 3 min, and the organic layer (bottom layer) was collected and evaporated under a gentle stream of nitrogen at 35 °C. The residues of the lipid derivatives were stored at −80 °C or immediately used for the measurement. The samples were dissolved in 250 μL of mixture MeOH/CHCl_3_ (1:1, v/v) just before the RP-UHPLC/MS/MS analysis. An overview of the sample preparation is illustrated in Figure 1.

### RP-UHPLC/MS/MS Conditions

The RP-UHPLC/MS/MS measurements were performed on an Agilent 1290 Infinity series liquid chromatograph (Agilent Technologies, Waldbronn, Germany) coupled to a Sciex QTRAP 6500 mass spectrometer (SCIEX, Framingham, MA, USA). The initial conditions for the separation method were inspired by our previous work^34^ with further optimization, and the final RP-UHPLC method used the following optimized conditions: Acquity UPLC BEH (Bridged Ethyl Hybrid) C18 column (150 × 2.1 mm, 1.7 μm), flow rate 0.35 mL/min, injection volume 2.5 μL, column temperature 55 °C, and autosampler temperature 4 °C. The linear gradient elution was set: 0 min – 35% B; 15 min – 80% B; 16 min – 90% B; 19 min – 90% B; 20 min – 35% B, where phase A was CH_3_CN/H_2_O (6:4, v/v), phase B was i-PrOH/CH_3_CN (9:1, v/v), and both phases contained 5 mM ammonium formate and 0.1% formic acid. The post-time was set to 2 min. The needle wash program runs from 16 min after each injection, including external wash by flash port and internal wash using drawing and ejection of the maximum volume of i-PrOH/MeOH/CHCl_3_ (4:2:1, v/v/v) + 5% H_2_O. The injector is switched to bypass during the needle wash program.

The mass spectrometer was equipped with a Turbo V ion source, and measurements were performed in negative ion ESI mode. The following optimized instrument settings were used: capillary voltage −4.5 kV, drying temperature 600 °C, curtain gas pressure 10 psi (69 KPa), nebulizer gas pressure 70 psi (483 KPa), heating gas pressure 70 psi (483 KPa), and acquisition *m*/*z* range 100–1000 with a scan time of 0.5 s. The collision energy (CE) and declustering potential (DP) were optimized for individual lipid classes based on the behavior of standards. All optimized parameters are summarized in Tables S4 and S5. To avoid contamination of the ionization source, a divert valve bypassing the ion source of the mass spectrometer was directed to waste during the interval of 16–20 min.

### Data Processing

All data were acquired using Analyst software (version 1.6.2) from SCIEX. Skyline software^36^ was used for the determination of the peak area of individual lipids during the optimization. The identification was performed manually using MRM scans, precursor ion scans (PIS), and neutral loss scans (NLS) based on characteristic fragment ions with confirmation by retention dependencies. The in-house database of lipids was used for identification.

## Results and Discussion

### Study Design

Although the concentrations of neutral lipids in biological samples are high,^37^ there is still a problem with the detection and accurate determination of the concentrations of these lipids by MS methods, especially sterols and their esters.^13,21^ Here, we focus on the analysis of mono- and diacylglycerols, free sterols with one hydroxyl group, and tocopherols, allowing the reaction with free hydroxyl group(s). Triacylglycerols and sterol esters can be hydrolyzed and derivatized as well, but then we would lose the natural profile of the sample, and therefore, we did not follow this way. Methods without and with the derivatization use the positive ion mode MS in almost all cases, which brings many issues in their analysis.^13^ For this reason, our idea was to use a derivatization reaction using the charge-switch to the negative ion mode, in which in-source fragmentation is significantly lower, and a smaller number of adducts is provided, leading to higher sensitivity. Several potential candidates for new derivatization agents were tested, such as pyridine-3-sulfonyl chloride, 4-(aminosulfonyl)benzoyl chloride, and 4-(dimethylamino)benzoyl chloride, but 3-(chlorosulfonyl)benzoic acid showed the greatest potential for our target (best MS response in negative ion mode) and therefore was selected for future optimization.

### Structural Characterization of Derivatives

**Figure 2:**
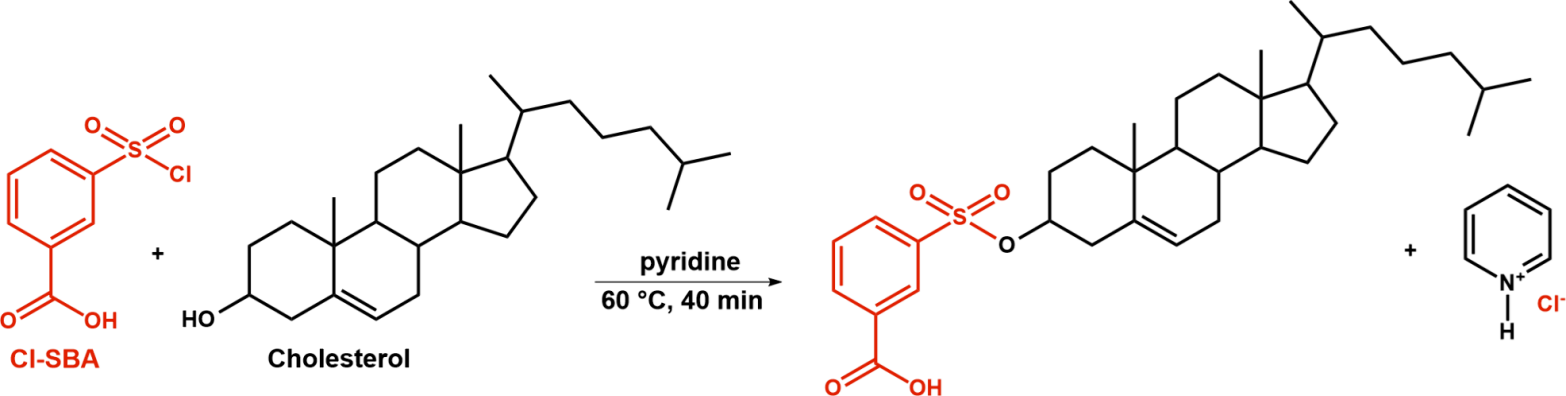
Reaction mechanism of derivatization using 3-(chlorosulfonyl)benzoic acid is shown for cholesterol.

The free hydroxyl group(s) reacts with the derivatization agent in the presence of pyridine to form a sulfonic ester (Figure 2). The basic environment is essential to adjust pH and neutralization of the formed acid during the reaction. To avoid unwanted hydrolysis of the derivatization agent, acetonitrile was used as reaction media. There is one reaction site for free sterols, tocopherols, and DG that leads to one reaction product, but MG can react twice, which can lead to two products (Figure S1). Lipid standards MG 18:1, DG 36:2, cholesterol D7, cholecalciferol, and a group of tocopherols (α-, γ-, δ-) were selected as representatives of individual lipid classes and used for preliminary experiments. All derivatives provide one monosubstituted product, except for MG producing both monosubstituted and disubstituted forms. All derivatives were detected in the form of deprotonated molecules and were singly charged. The fragmentation behavior was investigated by a high-resolution mass spectrometer (quadrupole–time-of-flight). Fragment ions provided by the derivatization agent were detected at *m*/*z* 200.99 ([C_7_H_5_O_5_S]^−^) and *m*/*z* 155.98 ([C_6_H_4_O_3_S]^−^) for all derivatives, except for tocopherols with a missing fragment [C_7_H_5_O_5_S]^−^. For MG and DG, neutral losses of fatty acyls ([M – 282.26]^−^ for C18:1) and the loss of sulfobenzoic acid ([M – 201.99]^−^) for disubstituted MG were also observed. Furthermore, the selective fragment at *m*/*z* 347.05 ([C_17_H_15_O_6_S]^−^, derivatized phenolic part) was detected for α-tocopherol and analogues for other tocopherols. MS/MS spectra of individual derivatives are shown in Figure S2.

### Optimization of Derivatization

Several parameters were investigated to obtain a high yield of the reaction, such as the molar ratio of pyridine and Cl-SBA (0:1, 1:1, 2:1, 4:1, and 6:1), the concentration of derivatization agent (10, 50, 100, and 150 mg/mL), the reaction temperature (20, 40, and 60 °C), and the reaction time (5, 10, 20, 40, and 60 min). The derivatization reaction was optimized by using pooled plasma spiked by 30 μL of the IS-Mix. Before derivatization, the proteins present in human plasma were eliminated by protein precipitation^5,34^ because free reaction sites can potentially increase the reagent amount needed for the derivatization. The optimization of the derivatization procedure is illustrated in Figures S3–S6 for all internal standards.

First, the molar ratio (v/v) of pyridine and 3-(chlorosulfonyl)benzoic acid at a constant concentration of 50 mg/mL in CH_3_CN of both was investigated. If no pyridine was used, the reaction yield was minimal and nonreproducible for all investigated lipid classes. Otherwise, the effect of amount of pyridine is different based on lipid class, but IS from the same lipid class follow the same trend (no effect of the length of acyl chain or the number of DB). The molar ratio 1:1 shows the best yield only for monosubstituted MG, while a higher amount of base increases formation of disubstituted MG, and the molar ratio 4:1 gives the best results. However, the best yield for almost all lipid classes shows the molar ratio 6:1, which was selected for the following optimization. Second, the concentrations of the derivatization agent were tested, while the molar ratio of pyridine and Cl-SBA 6:1 and the same total volume of reaction mixture (500 μL) for all experiments were maintained. The lower amount of derivatization agent (10 mg/mL) leads to an incomplete reaction and a higher production of monosubstituted MG, while the higher concentration increases the yield of the disubstituted form of MG. For concentrations of 50–150 mg/mL of the derivatization agent, comparable reaction yields were observed for standards from other lipid classes, but 50 mg/mL showed the most reproducible results. The last optimized parameters were reaction time and reaction temperature, which are closely related because at a lower reaction temperature, a longer reaction time is required and vice versa. If the reaction mixture is heated to 60 °C, the reaction is complete within 40 min, but at room temperature (20 °C), the reaction takes 60 min with a comparable yield (data not shown). Similarly to the previous cases, the optimal parameters for individual lipid classes are different. The short reaction time at room temperature is optimal for monosubstituted MG, while a higher reaction time and temperature are preferred for other lipid classes. The reaction of DG and disubstituted MG is already complete in 20 min at 60 °C, but due to other classes of lipids (free sterols and tocopherols), the optimal reaction time was set at 40 min. On the other hand, the temperature of the reaction can be critical for some analytes leading to degradation or generation of artifacts, but none was detected for the investigated lipid species. The reaction yield cannot be exactly calculated without synthesized derivatives of IS, but no residues of the natural forms of MG, DG, and free sterols were detected, indicating high derivatization efficiency.

The reaction is stopped by the addition of water and the protic solvent used in the Folch lipid extraction, leading to the hydrolysis of excessive derivatization reagent. However, the Folch extraction mainly reduces the excess of derivatization agent and pyridine, which can lead to contamination of the mass spectrometer. The acidic, neutral, and basic composition of the aqueous phase of the liquid–liquid extraction was investigated. The 0.1% water solution of formic acid, the original procedure of the Folch extraction^35^ with pure water, and 250 mM water solution of ammonium carbonate^38^ were compared. None of the tested aqueous phase compositions significantly reduce the signal of the monitored lipids (Figure S7), except under basic conditions in the case of the DG 33:1 D7 derivative. However, the neutral aqueous phase shows the highest extraction yield and reproducibility for almost all lipid classes.

### Stability of Derivatives

The stability of the analytes is important for reproducible measurements of larger sample sets to receive data of high quality. For this reason, the short- and long-term stabilities of derivatives were investigated. The pooled sample of derivatized human plasma spiked by IS-Mix to obtain the representative sample was prepared and subsequently aliquoted into individual vials. The samples used for long-term stability were stored at −80 °C, while others were measured on the same day. The short-term stability was performed in the autosampler set at 4 °C, when individual samples were placed in the autosampler and analyzed five times every 2 h, which simulates 10 h of continuous measurement. The results (Figure S8) show comparable values for all time points with RSD less than 10%, which indicates short-term stability at 4 °C for at least 10 h for all investigated derivatives. The long-term stability was tested for 5 days, when the first time point was analyzed immediately after the derivatization procedure, and other aliquots were stored at −80 °C. On subsequent days, samples were taken from the freezer and measured shortly after tempering to ambient temperature (each sample was exposed only to one freeze/thaw cycle). All time points show comparable results (Figure S9), with RSD less than 5% for all investigated derivatives, indicating excellent stability of the derivatives for at least 5 days stored at −80 °C. Since no significant decrease in response was observed during short- and long-term stabilities, the stability of derivatives could be much longer, but it was not tested.

### Optimization of RP-UHPLC/MS/MS Method

A reversed-phase UHPLC was used for the separation of derivatives, which allowed the resolution of individual isomers. The slope of the gradient was optimized using the derivatized standard mixture (STD-Mix) of lipids (Table S1). Compared to the natural form of lipids, derivatives are less retained and the higher number of derivatized sites significantly decrease retention time; for example, monosubstituted MG 18:1 elutes in 3.0 min, while disubstituted MG 18:1 elutes in 1.7 min. The isocratic part of the gradient at the end with high percentage of mobile phase B is due to the removal of nonderivatized nonpolar lipids (triacylglycerols and cholesteryl esters) from the column.

**Figure 3:**
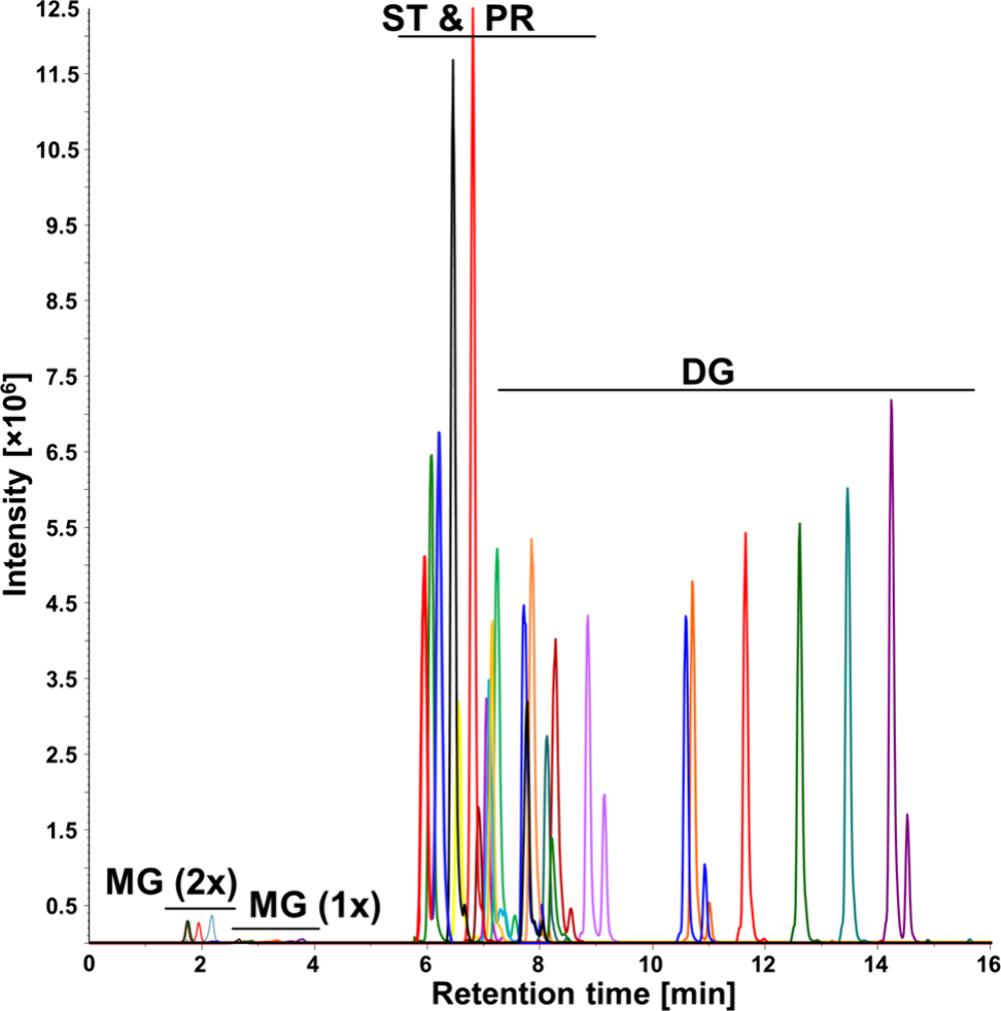
Extracted ion chromatogram of the derivatized standard mixture measured by the RP-UHPLC/MS/MS method. Individual standards are racemic mixtures, which lead to double peaks for individual standards in the chromatogram. Annotation: monoacylglycerol (MG), diacylglycerol (DG), sterol (ST), and prenol (PR).

UHPLC was connected to triple quadrupole MS, and the optimization of the MS conditions was performed using derivatized STD-Mix. All derivatives were measured in the negative ion mode and optimized parameters (nebulizer gas, heating gas, curtain gas, and source temperature) with the investigated ranges summarized in Table S4 and visualized in Figure S10. Drying temperature 600 °C, curtain gas pressure 10 psi, nebulizer gas pressure 70 psi, and heating gas pressure 70 psi provide the best MS signal response, and all derivatives follow the same trend. Collision energy and declustering potential were optimized by using derivatized standards, and optimal values for individual lipid classes are summarized in Table S5. The extracted ion chromatogram of the derivatized standard mixture that includes 29 lipid standards from 4 lipid classes measured by optimized RP-UHPLC/ESI-MS is shown in Figure 3 (more details of the chromatogram are shown in Figure S11). Individual standards are a racemic mixture, such as 1,2- and 1,3-DG, which leads to double peaks for individual standards in the chromatogram. 1,2-DG are eluted earlier than 1,3-DG (Figure S11D), which is the opposite mechanism than for nonderivatized forms.^39,40^ Furthermore, the RP-UHPLC method separates isomeric forms of lipids, such as desmosterol vs 7-dehydrocholesterol that differ in the position of DB (ring B of the sterol skeleton vs aliphatic part) vs cholecalciferol (different structure; Figure S12A) or cholesterol D7 vs lathosterol (Figure S12B) that differ in the position of DB on the ring B of the sterol skeleton.

### Verification of the Derivatization Procedure

**Figure 4:**
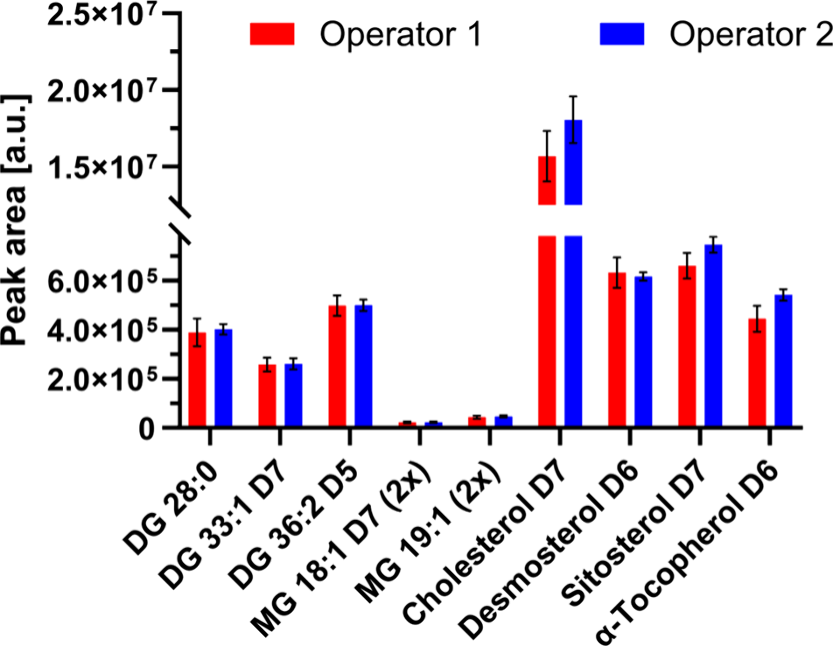
Reproducibility of the derivatization method investigated by two operators. Data are presented as the mean value ± the standard deviation from five independent experiments.

The derivatization method was verified by one and two operator(s). The reaction conditions were chosen based on suitability for most of the investigated lipid classes, which are not suitable for monosubstituted MG, and therefore, only disubstituted MG were further analyzed. The repeatability of the derivatization method was investigated by one operator who prepared 10 independent reactions with a relative standard deviation (RSD) lower than 11% for all lipid classes (Figure S13). Then, the reproducibility was investigated by two operators, each of 5 samples was independently prepared on the same day in the same laboratory according to the derivatization protocol. Figure 4 shows the comparable results between the operators, while the RSD of both operators was less than 13%, and the RSD of the more experienced operator was even less than 10%.

**Figure 5:**
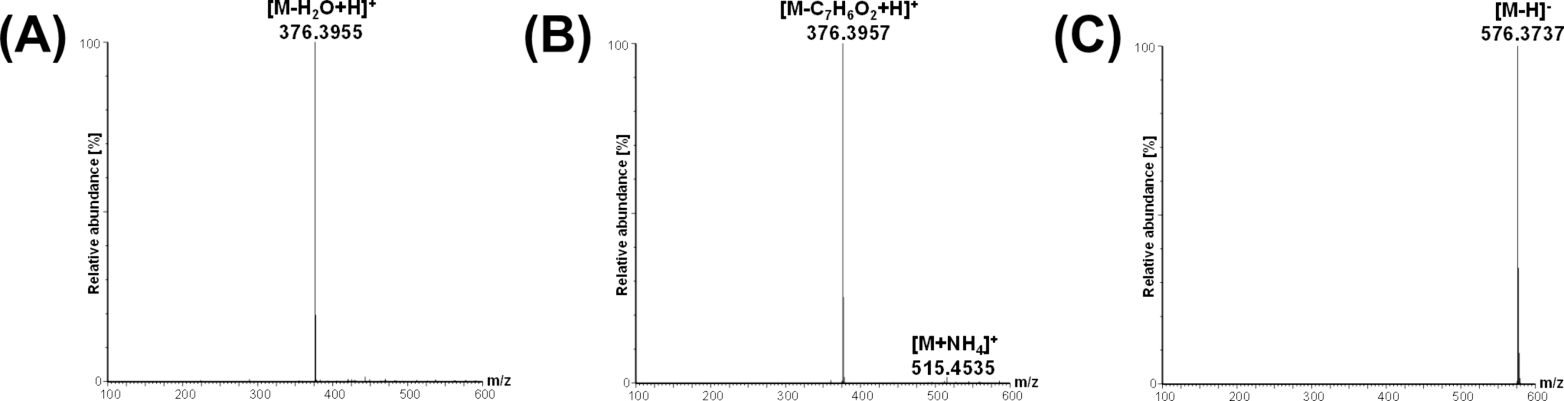
Comparison of in-source fragmentation of cholesterol D7 based on the analysis of (A) the nonderivatized form measured in the positive ion mode, (B) the benzoyl derivative^14^ measured in the positive ion mode, and (C) the SBA derivative measured in the negative ion mode. Spectra were measured by high-resolution mass spectrometer (Xevo G2-XS QTOF) using the same mass spectrometry conditions.^14^

The repeatability and reproducibility of the derivatization method were additionally verified at various concentration levels by using calibration curves. The pooled plasma (10 μL) was spiked with various volumes of IS-Mix before protein precipitation, and then the derivatization procedure was applied. Calibration curves were prepared based on the measurement of 15 concentration levels in triplicate, and the limits of detection (LOD), the limit of quantitation (LOQ), and the linear range were evaluated. Calibration curves (Figure S14) provide linear regression coefficients greater than 0.99 for all investigated analytes expect for α-tocopherol D6. LOQ expresses the concentration of IS that was determined with an accuracy error lower than ±20% and 2.5 nmol/mL of plasma for all investigated lipid classes were determined. LOD expresses the lowest detected concentration consistently and reliably, and 0.5 nmol/mL of plasma for α-tocopherol D6 and MG, 0.25 nmol/mL of plasma for DG, and 15–25 pmol/mL of plasma for free sterols were determined. All values are experimentally confirmed and not only theoretically calculated. The parameters for individual IS are summarized in Table S6. If the LOD is compared to our previous derivatization method,^14^ determining benzyol derivatives in the positive ion mode, the LODs for MG and DG were determined by a new method and are 2–3.5 times lower and for cholesterol D7 they are even lower by 3800 times. A comparison of LOD across the methods can be difficult because it can be calculated/determined in different ways, but here they compare the results from the same laboratory determined by the same procedure. The instrumentation can play important part for sensitivity of analysis (e.g., high-resolution and low-resolution MS), but the key aspect in this case is in-source fragmentation demonstrated on cholesterol D7, which the fragments are 100% for a nonderivatized form measured in the positive ion mode, 97% for the benzoyl derivative measured in the positive ion mode, and 0% (no in-source fragment was detected) for the new SBA-derivative (Figure 5).

### Identification of Lipids in Human Plasma

Finally, the optimized method was used for the identification of lipids from selected lipid classes in human plasma. The pooled human plasma sample was derivatized, and the characteristic PIS/NLS were used for the identification of features containing the derivatization tag, for which the product ion mass spectra were subsequently measured. Based on the behavior of standards, deprotonated molecules were detected for all analytes. The combination of MRM scans with retention dependencies^34^ leads to high confident identification. Generally, detected lipids can be annotated at various levels based on the obtained structural information. In our work, we distinguish three levels based on the information from MS/MS spectra and confirmation by standards: the species level representing the sum composition (sum of carbon atoms and DB), the molecular species level carrying information about the composition of fatty acyls, and the complete structure level for analytes confirmed by an identical standard. PIS of 201.0 allows the annotation of derivatives at the species level, but the NLS of fatty acids brings more detailed information for DG and allows for reporting at the molecular species level. The shorthand nomenclature for lipids is used according to Liebisch et al.^41^

The combination of reversed-phase UHPLC and MS allows resolution of various isomers, such as fatty acyl composition, *sn*-positions for DG, and position of double bond for sterols. Isomers with different *sn*-positions are separated by chromatography (retention times differ by ∼ 0.4 min). Otherwise, isomers with different fatty acyl compositions coelute together (Δ ∼ 0.1 min) but produce characteristic fragments in MS/MS. Furthermore, correct lipid annotation was confirmed based on retention dependences of retention times of lipids on the length of the fatty acyl chain/carbon number (Figure S15) and the number of the DB (Figure S16). The retention dependencies also confirmed the presence of two series of DG differing in the *sn*-position. An exception to retention dependence may appear to be lanosterol (ST 30:2;O), eluting significantly earlier than stigmasterol (ST 29:2;O), which is explained by different structures. Lanosterol contains two methyl groups linked to ring A compared to other detected sterols, which differ in the number of DB or composition of the aliphatic chains. For sterols, the retention dependencies are slightly complicated because there is an influence of structural differences and the position of the DB on the retention time leading to multiple homologous series in the graph. However, the retention time of most sterols was confirmed by standards, but there are still other standards, which can be used for extension of the identification.

In total, 92 derivatized lipid species from the 4 lipid classes (8 monoacylglycerols, 66 diacylglycerols, 15 free sterols, and 3 prenols) were detected in human plasma (Table S7 and Figure S17). Compared to published methods (Table 1) using nonderivatized and derivatized approaches for lipidomic analysis of human plasma/serum by LC/MS, LC/MS/MS, and SFC/MS methods, our new method enables the detection of more lipid species for all investigated lipid classes. We focused only on free sterols with one hydroxyl group, but some other methods investigated also the analysis of oxysterols,^47−49^ which are not included in the final comparison. Nevertheless, most of the methods focus on more complex lipidomic analysis, which can affect the sensitivity of the analysis, leading to a lower number of reported lipid species from these classes. However, especially the determination of free sterols without the derivatization is complicated, and only cholesterol is typically detected, regardless of the high concentration (770 nmol/mL^42^) in the human plasma. Other free sterols are present at significantly lower concentrations (100–1000× lower^43^), which is below the LOD for many methods, and special methods^47−52^ must be used for determination. Moreover, significantly higher cholesterol concentration results in a wider chromatographic peak, which can lead to the overlap of other isomers in one chromatographic peak compared to baseline separation optimized on standards. However, our chromatographic method demonstrated high separation efficiency also for the real sample, and 3 isomer forms of ST 27:1;O were clearly detected in human plasma visualized on Figure S17C. The M + 2 isotope of ST 27:1;O can lead to false identification and complicate the determination of another lipid species with two additional hydrogens (ST 27:0;O), but Figure S17C also demonstrates the elimination of the potential interference using chromatography separation. The analysis of DG and MG is a challenge as well because they cannot be determined by techniques like HILIC/MS and direct infusion MS due to coelution/co-ionization together with triacylglycerols producing identical in-source fragments. UHPSFC/MS seems to be a sensitive technique for the determination of these substances, but this technique is not very widespread yet.^10^ Especially for MG and free sterols, the combination of separation methods with high-resolution MS must be used due to the absence of fragments for PIS, NLS, and MRM, which are mainly used in low-resolution MS. However, our derivatization method combined with the RP-UHPLC/MS/MS method offers an easy, selective, and sensitive method without instrumental complexity for the analysis of these biologically interesting substances, which are important for the study of metabolic pathways.

**Table 1:** Comparison of the Number of Identified Lipid Species in the Human Plasma/Serum with the Literature^a^

lipid class	MG	DG	free ST	PR	sum
RP-UHPLC/MS/MS + new derivatization	8	66	15	3	92
RP-UHPLC/MS/MS^5^^,^^b^	0	20	1	1	22
RP-UHPLC/MS + derivatization^14^	5	40	1	0	46
RP-UHPLC/MS^34^	4	20	1	0	25
ring trial A^42^^,^^b^	0	24	1	0	25
ring trial B^43^^,^^b^	0	55	2 + 6^c^	2	65
ring trial C^44^^,^^b^	0	31	0	0	31
UHPSFC/MS^45^^,^^b^	3	22	1	1	27
UHPLC/TIMS-MS^46^	0	20	1	0	21
RP-UHPLC/MS^n^ + derivatization^47^			8		8
RP-HPLC/MS/MS^48^^,^^b^			7		7
RP-HPLC/MS/MS^49^^,^^b^			7		7
SPE-RP-HPLC/MS^50^^,^^b^			5		5
RP-HPLC/MS/MS^51^^,^^b^			9 + 1^c^		10
RP-HPLC/MS/MS + derivatization^52^^,^^b^			11		11

a Annotation: monoacylglycerol (MG), diacylglycerol (DG), sterol (ST), and prenol (PR).

b Method was applied for quantitative analysis.

c Determined by gas chromatography/mass spectrometry.

## Conclusions

We introduce a novel derivatization method using 3-(chlorosulfonyl)benzoic acid, which has never been used as a derivatization agent. The derivatization reaction brings a charge-switch to a negative ion mode for selected lipid classes, which are normally detected in the positive ion mode. This charge-switch leads to the high stability of analytes in the ion source, higher ionization efficiency, and the formation of diagnostic fragments for PIS, NLS, and MRM transitions. The derivatization reaction is simple because it involves mixing only reagents and reaction at a certain temperature for a given time, which facilitates potential implementation in other laboratories. Moreover, the high stability of the derivatives, reproducibility, and repeatability of the reaction were confirmed, which allows for the use of the reaction for routine analysis. The combination of derivatization and RP-UHPLC/MS/MS methods leads to a highly sensitive and selective analysis with a low LOD, especially for free sterols in the range of 15–25 pmol/mL of plasma. The application of complete methodology was demonstrated for the qualitative analysis of human plasma. In total, 8 monoacylglycerols, 66 diacylglycerols, 15 free sterols, and 3 prenols were detected, which is a significant improvement for the investigated lipid classes compared to the published methods. The validation of the derivatization approach and application for lipidomic quantitation in a large clinical cohort of cancer patients is the subject of our follow-up research.

## Supporting Information Available

Structure of derivatives; Tandem mass spectra; Optimization of methods; Extracted ion chromatograms; Verification of derivatization; Calibration curves; Retention dependecies (PDF)
Composition of standard and internal standard mixtures; Clinical information; Optimized parameters; Parameters determined from the calibration curves; List of identified lipids (XLSX)

## Notes

All volunteers signed an informed consent and the ethics committee approved the blood collection.

## References

[ref1] Han X. L. Lipidomics for studying metabolism. Nat. Rev. Endocrinol. 2016, 12 (11), 668–679. 10.1038/nrendo.2016.98. 27469345

[ref2] Yang X. K.; Liu L.; Xi L. J.; Wu B. B.; Ku C. Y.; Wang R. Z.; Dai M.; Ping Z. G. Trends in total cholesterol control among American adults with hypercholesterolemia, 1988–2018. Nutr. Metab. Cardiovas. 2023, 33 (8), 1511–1520. 10.1016/j.numecd.2023.05.015. 37344285

[ref3] Zambón D.; Quintana M.; Mata P.; Alonso R.; Benavent J.; Cruz-Sánchez F.; Gich J.; Pocoví M.; Civeira F.; Capurro S.; Bachman D.; Sambamurti K.; Nicholas J.; Pappolla M. A. Higher Incidence of Mild Cognitive Impairment in Familial Hypercholesterolemia. Am. J. Med. 2010, 123 (3), 267–274. 10.1016/j.amjmed.2009.08.015. 20193836 PMC2844655

[ref4] Singh M.; Nam D. T.; Arseneault M.; Ramassamy C. Role of By-Products of Lipid Oxidation in Alzheimer’s Disease Brain: A Focus on Acrolein. J. Alzheimers Dis. 2010, 21 (3), 741–756. 10.3233/JAD-2010-100405. 20634576

[ref5] Huynh K.; Barlow C. K.; Jayawardana K. S.; Weir J. M.; Mellett N. A.; Cinel M.; Magliano D. J.; Shaw J. E.; Drew B. G.; Meikle P. J. High-Throughput Plasma Lipidomics: Detailed Mapping of the Associations with Cardiometabolic Risk Factors. Cell Chem. Biol. 2019, 26 (1), 71–84. 10.1016/j.chembiol.2018.10.008. 30415965

[ref6] Wolrab D.; Jirásko R.; Chocholoušková M.; Peterka O.; Holčapek M. Oncolipidomics: Mass spectrometric quantitation of lipids in cancer research. Trac-Trend Anal. Chem. 2019, 120, 115480 10.1016/j.trac.2019.04.012.

[ref7] Avela H. F.; Sirén H. Advances in lipidomics. Clin. Chim. Acta 2020, 510, 123–141. 10.1016/j.cca.2020.06.049. 32622966

[ref8] Kanehisa M.; Furumichi M.; Sato Y.; Kawashima M.; Ishiguro-Watanabe M. KEGG for taxonomy-based analysis of pathways and genomes. Nucleic Acids Res. 2023, 51 (1), 587–592. 10.1093/nar/gkac963. PMC9825424 36300620

[ref9] Lange M.; Ni Z. X.; Criscuolo A.; Fedorova M. Liquid Chromatography Techniques in Lipidomics Research. Chromatographia. 2019, 82 (1), 77–100. 10.1007/s10337-018-3656-4.

[ref10] Wolrab D.; Peterka O.; Chocholoušková M.; Holčapek M. Ultrahigh-performance supercritical fluid chromatography/mass spectrometry in the lipidomic analysis. Trac-Trend Anal. Chem. 2022, 149, 116546 10.1016/j.trac.2022.116546.

[ref11] Holčapek M.; Liebisch G.; Ekroos K. Lipidomic Analysis. Anal. Chem. 2018, 90 (7), 4249–4257. 10.1021/acs.analchem.7b05395. 29543437

[ref12] Züllig T.; Trötzmüller M.; Köfeler H. C. Lipidomics from sample preparation to data analysis: a primer. Anal. Bioanal. Chem. 2020, 412 (10), 2191–2209. 10.1007/s00216-019-02241-y. 31820027 PMC7118050

[ref13] Murphy R. C. Challenges in mass spectrometry-based lipidomics of neutral lipids. Trac-Trend Anal. Chem. 2018, 107, 91–98. 10.1016/j.trac.2018.07.023. PMC6483396 31031456

[ref14] Peterka O.; Jirásko R.; Vaňková Z.; Chocholoušková M.; Wolrab D.; Kulhánek J.; Bureš F.; Holčapek M. Simple and Reproducible Derivatization with Benzoyl Chloride: Improvement of Sensitivity for Multiple Lipid Classes in RP-UHPLC/MS. Anal. Chem. 2021, 93 (41), 13835–13843. 10.1021/acs.analchem.1c02463. 34623138

[ref15] Li Y. L.; Su X.; Stahl P. D.; Gross M. L. Quantification of diacylglycerol molecular species in biological samples by electrospray ionization mass spectrometry after one-step derivatization. Anal. Chem. 2007, 79 (4), 1569–1574. 10.1021/ac0615910. 17297957 PMC2573952

[ref16] Leiker T. J.; Barkley R. M.; Murphy R. C. Analysis of diacylglycerol molecular species in cellular lipid extracts by normal-phase LC-electrospray mass spectrometry. Int. J. Mass Spectrom. 2011, 305 (2–3), 103–108. 10.1016/j.ijms.2010.09.008. 21860599 PMC3158596

[ref17] Liu Y. D.; Liu H. J.; Gong G. W. Monitoring diacylglycerols in biofluids by non-isotopically paired charge derivatization combined with LC-MS/MS. Front. Chem. 2022, 10, 1062118 10.3389/fchem.2022.1062118. 36523747 PMC9745812

[ref18] Petrosino T.; Riccieri R.; Blasi F.; Brutti M.; D’arco G.; Bosi A.; Maurelli S.; Cossignani L.; Simonetti M. S.; Damiani P. Original normal-phase high-performance liquid chromatographic separation of monoacylglycerol classes from extra virgin olive oil Triacylglycerols for their stereospecific analysis. J. Aoac. Int. 2007, 90 (6), 1647–1654. 10.1093/jaoac/90.6.1647. 18193743

[ref19] Tada N.; Fujita H.; Ando Y. Synthesis of Urethane Derivatives of Mono- and Diacylglycerols for Use as HPLC Standards in the Enantiomeric Separation. J. Am. Oil Chem. Soc. 2014, 91 (7), 1131–1137. 10.1007/s11746-014-2452-z.

[ref20] Zhu M. L.; Lu K. G.; Jin Y. T.; Xu X. W.; Chu C. Y.; Hao H. P.; Zheng Q. L. Boronic derivatization-based strategy for monoacylglycerol identification, isomer annotation and quantification. Anal. Chim. Acta 2022, 1190, 339233 10.1016/j.aca.2021.339233. 34857145

[ref21] Liebisch G.; Binder M.; Schifferer R.; Langmann T.; Schulz B.; Schmitz G. High throughput quantification of cholesterol and cholesteryl ester by electrospray ionization tandem mass spectrometry (ESI-MS/MS). Bba-Mol. Cell Biol. 2006, 1761 (1), 121–128. 10.1016/j.bbalip.2005.12.007. 16458590

[ref22] Nzekoue F. K.; Caprioli G.; Ricciutelli M.; Cortese M.; Alesi A.; Vittori S.; Sagratini G. Development of an innovative phytosterol derivatization method to improve the HPLC-DAD analysis and the ESI-MS detection of plant sterols/stanols. Food Res. Int. 2020, 131, 108998 10.1016/j.foodres.2020.108998. 32247468

[ref23] Woo H. K.; Go E. P.; Hoang L.; Trauger S. A.; Bowen B.; Siuzdak G.; Northen T. R. Phosphonium labeling for increasing metabolomic coverage of neutral lipids using electrospray ionization mass spectrometry. Rapid Commun. Mass Sp. 2009, 23 (12), 1849–1855. 10.1002/rcm.4076. PMC3052201 19449318

[ref24] Sandhoff R.; Brügger B.; Jeckel D.; Lehmann W. D.; Wieland F. T. Determination of cholesterol at the low picomole level by nano-electrospray ionization tandem mass spectrometry. J. Lipid Res. 1999, 40 (1), 126–132. 10.1016/S0022-2275(20)33347-2. 9869658

[ref25] Ayciriex S.; Regazzetti A.; Gaudin M.; Prost E.; Dargère D.; Massicot F.; Auzeil N.; Laprévote O. Development of a novel method for quantification of sterols and oxysterols by UPLC-ESI-HRMS: application to a neuroinflammation rat model. Anal. Bioanal. Chem. 2012, 404 (10), 3049–3059. 10.1007/s00216-012-6396-6. 23010846

[ref26] Honda A.; Yamashita K.; Miyazaki H.; Shirai M.; Ikegami T.; Xu G. R.; Numazawa M.; Hara T.; Matsuzaki Y. Highly sensitive analysis of sterol profiles in human serum by LC-ESI-MS/MS. J. Lipid Res. 2008, 49 (9), 2063–2073. 10.1194/jlr.D800017-JLR200. 18503032

[ref27] Jiang X. T.; Sidhu R.; Porter F. D.; Yanjanin N. M.; Speak A. O.; Vruchte D. T. T.; Platt F. M.; Fujiwara H.; Scherrer D. E.; Zhang J.; Dietzen D. J.; Schaffer J. E.; Ory D. S. A sensitive and specific LC-MS/MS method for rapid diagnosis of Niemann-Pick C1 disease from human plasma. J. Lipid Res. 2011, 52 (7), 1435–1445. 10.1194/jlr.D015735. 21518695 PMC3122908

[ref28] Griffiths W. J.; Crick P. J.; Wang Y. C.; Ogundare M.; Tuschl K.; Morris A. A.; Bigger B. W.; Clayton P. T.; Wang Y. Q. Analytical strategies for characterization of oxysterol lipidomes: Liver X receptor ligands in plasma. Free Radical. Bio. Med. 2013, 59, 69–84. 10.1016/j.freeradbiomed.2012.07.027. 22846477

[ref29] Roberg-Larsen H.; Strand M. F.; Grimsmo A.; Olsen P. A.; Dembinski J. L.; Rise F.; Lundanes E.; Greibrokk T.; Krauss S.; Wilson S. R. High sensitivity measurements of active oxysterols with automated filtration/filter backflush-solid phase extraction-liquid chromatography-mass spectrometry. J. Chromatogr. A 2012, 1255, 291–297. 10.1016/j.chroma.2012.02.002. 22410154

[ref30] Zhang W. P.; Zhang D. H.; Chen Q. H.; Wu J. H.; Ouyang Z.; Xia Y. Online photochemical derivatization enables comprehensive mass spectrometric analysis of unsaturated phospholipid isomers. Nat. Commun. 2019, 10 (1), 79 10.1038/s41467-018-07963-8. 30622271 PMC6325166

[ref31] Poad B. L. J.; Zheng X. Y.; Mitchell T. W.; Smith R. D.; Baker E. S.; Blanksby S. J. Online Ozonolysis Combined with Ion Mobility-Mass Spectrometry Provides a New Platform for Lipid Isomer Analyses. Anal. Chem. 2018, 90 (2), 1292–1300. 10.1021/acs.analchem.7b04091. 29220163 PMC5771865

[ref32] Deng P.; Zhong D. F.; Wang X.; Dai Y. L.; Zhou L.; Leng Y.; Chen X. Y. Analysis of diacylglycerols by ultra performance liquid chromatography-quadrupole time-of-flight mass spectrometry: Double bond location and isomers separation. Anal. Chim. Acta 2016, 925, 23–33. 10.1016/j.aca.2016.04.051. 27188314

[ref33] Feng Y.; Chen B. M.; Yu Q. Y.; Li L. J. Identification of Double Bond Position Isomers in Unsaturated Lipids by m-CPBA Epoxidation and Mass Spectrometry Fragmentation. Anal. Chem. 2019, 91 (3), 1791–1795. 10.1021/acs.analchem.8b04905. 30608661 PMC6408215

[ref34] Vaňková Z.; Peterka O.; Chocholoušková M.; Wolrab D.; Jirásko R.; Holčapek M. Retention dependences support highly confident identification of lipid species in human plasma by reversed-phase UHPLC/MS. Anal. Bioanal. Chem. 2022, 414 (1), 319–331. 10.1007/s00216-021-03492-4. 34244835

[ref35] Folch J.; Lees M.; Stanley G. H. S. A Simple Method for the Isolation and Purification of Total Lipides from Animal Tissues. J. Biol. Chem. 1957, 226, 497–509. 10.1016/S0021-9258(18)64849-5. 13428781

[ref36] Adams K. J.; Pratt B.; Bose N.; Dubois L. G.; St John-Williams L.; Perrott K. M.; Ky K.; Kapahi P.; Sharma V.; MacCoss M. J.; Moseley M. A.; Colton C. A.; MacLean B. X.; Schilling B.; Thompson J. W. Skyline for Small Molecules: A Unifying Software Package for Quantitative Metabolomics. J. Proteome Res. 2020, 19 (4), 1447–1458. 10.1021/acs.jproteome.9b00640. 31984744 PMC7127945

[ref37] Burla B.; Arita M.; Arita M.; Bendt A. K.; Cazenave-Gassiot A.; Dennis E. A.; Ekroos K.; Han X. L.; Ikeda K.; Liebisch G.; Lin M. K.; Loh T. P.; Meikle P. J.; Oresic M.; Quehenberger O.; Shevchenko A.; Torta F.; Wakelam M. J. O.; Wheelock C. E.; Wenk M. R. MS-based lipidomics of human blood plasma: a community-initiated position paper to develop accepted guidelines. J. Lipid Res. 2018, 59 (10), 2001–2017. 10.1194/jlr.S087163. 30115755 PMC6168311

[ref38] Wolrab D.; Chocholoušková M.; Jirásko R.; Peterka O.; Holčapek M. Validation of lipidomic analysis of human plasma and serum by supercritical fluid chromatography-mass spectrometry and hydrophilic interaction liquid chromatography-mass spectrometry. Anal. Bioanal. Chem. 2020, 412 (10), 2375–2388. 10.1007/s00216-020-02473-3. 32078000

[ref39] Holčapek M.; Jandera P.; Fischer J. Analysis of acylglycerols and methyl esters of fatty acids in vegetable oils and in biodiesel. Crit. Rev. Anal. Chem. 2001, 31 (1), 53–56. 10.1080/20014091076686.

[ref40] Saberi A. H.; Chin-Ping T.; Kee B. B.; Koon L. S.; Oi-Ming L. Reversed-Phase High-Performance Liquid Chromatography Analysis of 1,3-and 1,2(2,3)-Positional Isomers of Palm-Based Diacylglycerols. J. Oil Palm Res. 2013, 25 (3), 326–335.

[ref41] Liebisch G.; Fahy E.; Aoki J.; Dennis E. A.; Durand T.; Ejsing C. S.; Fedorova M.; Feussner I.; Griffiths W. J.; Köfeler H.; Merrill A. H.; Murphy R. C.; O’Donnell V. B.; Oskolkova O.; Subramaniam S.; Wakelam M. J. O.; Spener F. Update on LIPID MAPS classification, nomenclature, and shorthand notation for MS-derived lipid structures. J. Lipid Res. 2020, 61 (12), 1539–1555. 10.1194/jlr.S120001025. 33037133 PMC7707175

[ref42] Bowden J. A.; Heckert A.; Ulmer C. Z.; Jones C. M.; Koelmel J. P.; Abdullah L.; Ahonen L.; Alnouti Y.; Armando A. M.; Asara J. M.; Bamba T.; Barr J. R.; Bergquist J.; Borchers C. H.; Brandsma J.; Breitkopf S. B.; Cajka T.; Cazenave-Gassiot A.; Checa A.; Cinel M. A.; et al. Harmonizing lipidomics: NIST interlaboratory comparison exercise for lipidomics using SRM 1950-Metabolites in Frozen Human Plasma. J. Lipid Res. 2017, 58 (12), 2275–2288. 10.1194/jlr.M079012. 28986437 PMC5711491

[ref43] Quehenberger O.; Armando A. M.; Brown A. H.; Milne S. B.; Myers D. S.; Merrill A. H.; Bandyopadhyay S.; Jones K. N.; Kelly S.; Shaner R. L.; Sullards C. M.; Wang E.; Murphy R. C.; Barkley R. M.; Leiker T. J.; Raetz C. R. H.; Guan Z. Q.; Laird G. M.; Six D. A.; Russell D. W.; et al. Lipidomics reveals a remarkable diversity of lipids in human plasma. J. Lipid Res. 2010, 51 (11), 3299–3305. 10.1194/jlr.M009449. 20671299 PMC2952570

[ref44] Ghorasaini M.; Mohammed Y.; Adamski J.; Bettcher L.; Bowden J. A.; Cabruja M.; Contrepois K.; Ellenberger M.; Gajera B.; Haid M.; Hornburg D.; Hunter C.; Jones C. M.; Klein T.; Mayboroda O.; Mirzaian M.; Moaddel R.; Ferrucci L.; Lovett J.; Nazir K.; et al. Cross-Laboratory Standardization of Preclinical Lipidomics Using Differential Mobility Spectrometry and Multiple Reaction Monitoring. Anal. Chem. 2021, 93 (49), 16369–16378. 10.1021/acs.analchem.1c02826. 34859676 PMC8674878

[ref45] Wolrab D.; Jirásko R.; Cífková E.; Höring M.; Mei D.; Chocholoušková M.; Peterka O.; Idkowiak J.; Hrnciarová T.; Kuchař L.; Ahrends R.; Brumarová R.; Friedecký D.; Vivo-Truyols G.; Skrha P.; Škrha J.; Kučera R.; Melichar B.; Liebisch G.; Burkhardt R.; et al. Lipidomic profiling of human serum enables detection of pancreatic cancer. Nat. Commun. 2022, 13 (1), 124 10.1038/s41467-021-27765-9. 35013261 PMC8748654

[ref46] Lerner R.; Baker D.; Schwitter C.; Neuhaus S.; Hauptmann T.; Post J. M.; Kramer S.; Bindila L. Four-dimensional trapped ion mobility spectrometry lipidomics for high throughput clinical profiling of human blood samples. Nat. Commun. 2023, 14 (1), 937 10.1038/s41467-023-36520-1. 36806650 PMC9941096

[ref47] Teng Y. C.; Gielen M. C.; de Gruijter N. M.; Ciurtin C.; Rosser E. C.; Karu K. Phytosterols in human serum as measured using a liquid chromatography tandem mass spectrometry. J. Steroid Biochem. 2024, 241, 106519 10.1016/j.jsbmb.2024.106519. 38614432

[ref48] Ceglarek U.; Dittrich J.; Leopold J.; Helmschrodt C.; Becker S.; Staab H.; Richter O.; Rohm S.; Aust G. Free cholesterol, cholesterol precursor and plant sterol levels in atherosclerotic plaques are independently associated with symptomatic advanced carotid artery stenosis. Atherosclerosis. 2020, 295, 18–24. 10.1016/j.atherosclerosis.2019.12.018. 31981947

[ref49] Baila-Rueda L.; Cenarro A.; Cofán M.; Orera I.; Barcelo-Batllori S.; Pocoví M.; Ros E.; Civeira F.; Nerín C.; Domeño C. Simultaneous determination of oxysterols, phytosterols and cholesterol precursors by high performance liquid chromatography tandem mass spectrometry in human serum. Anal. Methods-Uk. 2013, 5 (9), 2249–2257. 10.1039/c3ay26395a.

[ref50] Mendiara I.; Bentayeb K.; Nerín C.; Domeño C. Online solid-phase extraction-liquid chromatography-mass spectrometry to determine free sterols in human serum. Talanta. 2015, 132, 690–697. 10.1016/j.talanta.2014.10.029. 25476366

[ref51] McDonald J. G.; Smith D. D.; Stiles A. R.; Russell D. W. A comprehensive method for extraction and quantitative analysis of sterols and secosteroids from human plasma. J. Lipid Res. 2012, 53 (7), 1399–1409. 10.1194/jlr.D022285. 22517925 PMC3371252

[ref52] Honda A.; Yamashita K.; Miyazaki H.; Shirai M.; Ikegami T.; Xu G. R.; Numazawa M.; Hara T.; Matsuzaki Y. Highly sensitive analysis of sterol profiles in human serum by LC-ESI-MS/MS. J. Lipid Res. 2008, 49 (9), 2063–2073. 10.1194/jlr.D800017-JLR200. 18503032

