# Supplementary material for: Novel Charge-Switch Derivatization Method Using 3-(Chlorosulfonyl)benzoic Acid for Sensitive RP-UHPLC/MS/MS Analysis of Acylglycerols, Sterols, and Prenols
Source: Anal Chem. 2025 Mar 28;97(13):7157–64. doi: 10.1021/acs.analchem.4c06496 (PMC11983369; doi:10.1021/acs.analchem.4c06496)

SUPPORTING INFORMATION

Novel Charge-Switch Derivatization Method Using 3-(Chlorosulfonyl)benzoic Acid for Sensitive RP-UHPLC/MS/MS Analysis of Acylglycerols, Sterols, and Prenols

Ondřej Peterka[1,#], Yasmin Kadyrbekova[1,#], Robert Jirásko[1], Zuzana Lásko[1], Bohuslav Melichar[2], Michal Holčapek[1,*]

[1] University of Pardubice, Faculty of Chemical Technology, Department of Analytical Chemistry, Studentská 573, 532 10 Pardubice, Czech Republic

[2] Palacký University Medical School and University Hospital Olomouc, Faculty of Medicine and Dentistry, Department of Oncology, I.P. Pavlova 6, 775 20 Olomouc, Czech Republic

Author contributions:

[#] O.P. and Y.K. contributed equally to this work.

Corresponding author:

Michal Holčapek, Tel. +420466037087; Email: michal.holcapek@upce.cz

Table of content

Figure S1: General chemical structures of derivatives representing 3
Figure S2: Tandem mass spectra of derivatized standards...4
Figure S3: Effect of molar ratio (*v/v*) of pyridine and 3-(chlorosulfonyl)benzoic acid (Cl-SBA) on the MS signal response of studied lipid derivatives...6
Figure S4: Effect of concentration of derivatization agent in acetonitrile (mg/mL) on the MS signal response of studied lipid derivatives 7
Figure S5: Effect of reaction temperature (°C) on the MS signal response of studied lipid derivatives...8
Figure S6: Effect of reaction time at 60 °C (min) on the MS signal response of studied lipid derivatives...9
Figure S7: Optimization of composition of aqua phase for Folch extraction.....................10
Figure S8: Short-term stability test investigated stability of derivates.............................. 11
Figure S9: Long-term stability test investigated stability..12
Figure S10: Optimization of mass spectrometry parameters using standard mix............13
Figure S11: Extracted ion chromatograms of the derivatized standard 14
Figure S12: Extracted ion chromatograms of the derivatized isomeric sterols. 16
Figure S13: Repeatability of derivatization method investigated by spiked human plasma 17
Figure S14: Calibration curves of derivatized internal standards in spiked human plasma 18
Figure S15: Graphical visualization of dependencies of the retention time on the carbon number..19
Figure S16: Graphical visualization of dependencies of the retention time on number of double bond(s) 20
Figure S17: Chromatograms of derivatized plasma sample. 21

Figure S1: General chemical structures of derivatives representing: **(A)** monoacylglycerols monosubstituted, **(B)** monoacylglycerols disubstituted, **(C)** diacylglycerols, **(D)** cholesterol, **(E)** cholecalciferol, and **(F)** α-tocopherol.

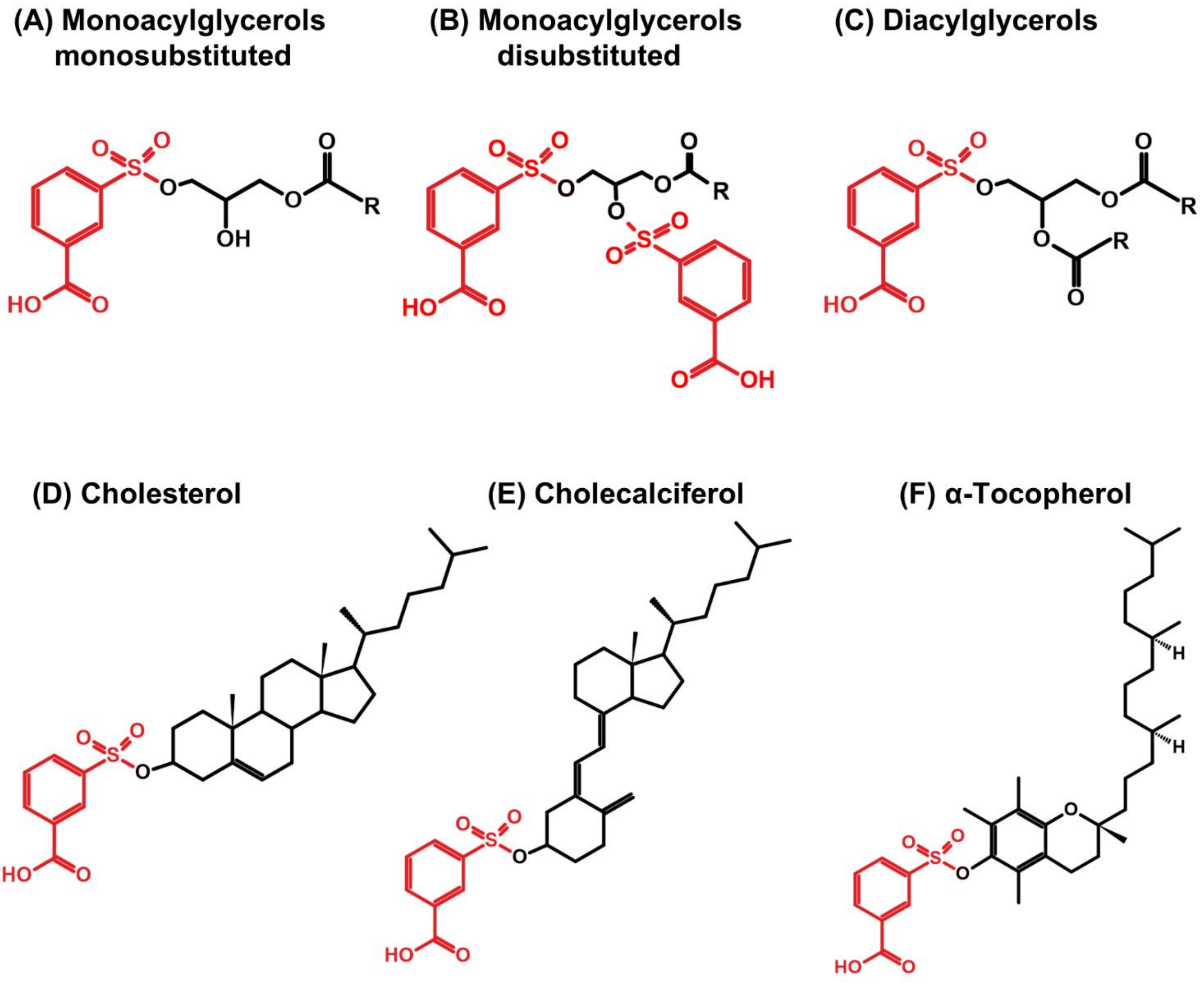

Figure S2: Tandem mass spectra of derivatized standards measured by high resolution mass spectrometer (Xevo G2-XS hybrid quadrupole – time of flight) using collision energy 30 eV: **(A)** monoacylglycerol 18:1 (1x), **(B)** monoacylglycerol 18:1 (2x), **(C)** diacylglycerol 18:1/18:1, **(D)** cholesterol D7, **(E)** cholecalciferol, **(F)** α-tocopherol, **(G)** δ- tocopherol, **(H)** γ-tocopherol.

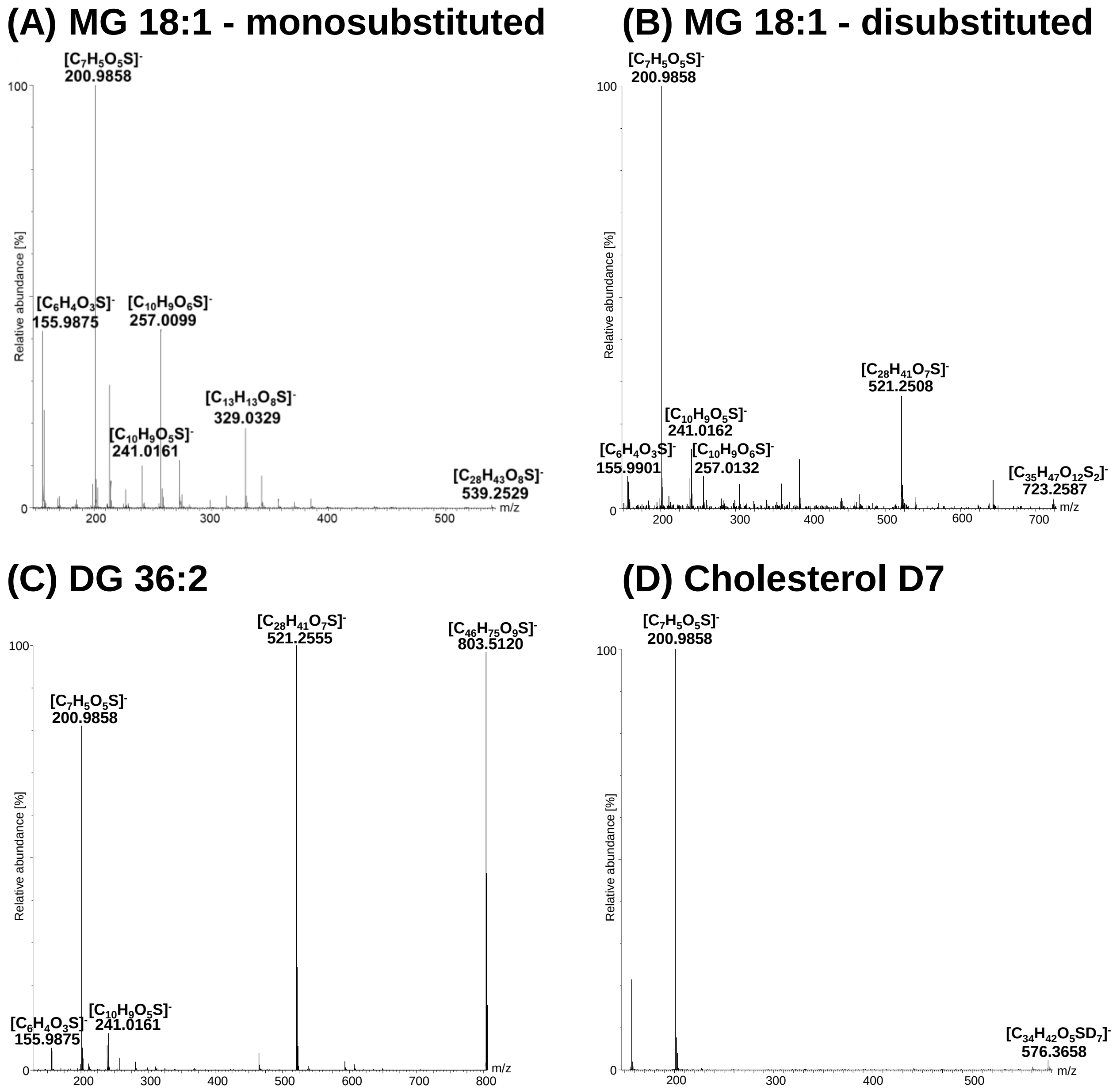

(E) Cholecalciferol

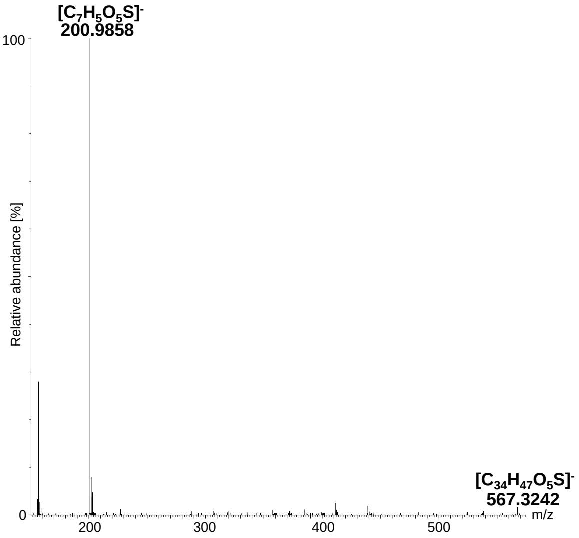

(F) α-tocopherol

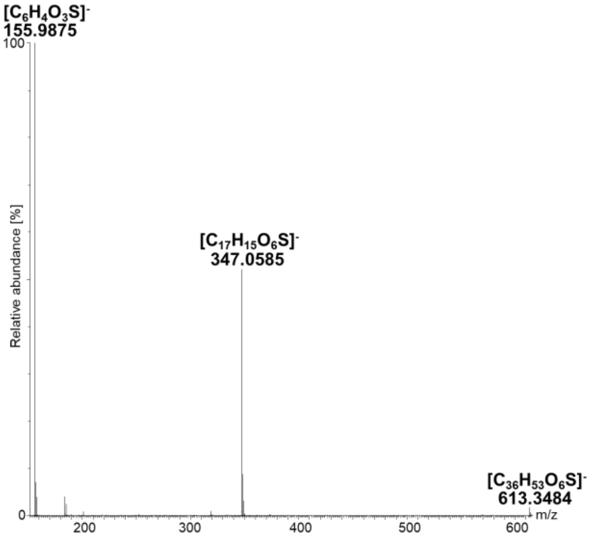

(G) δ-tocopherol

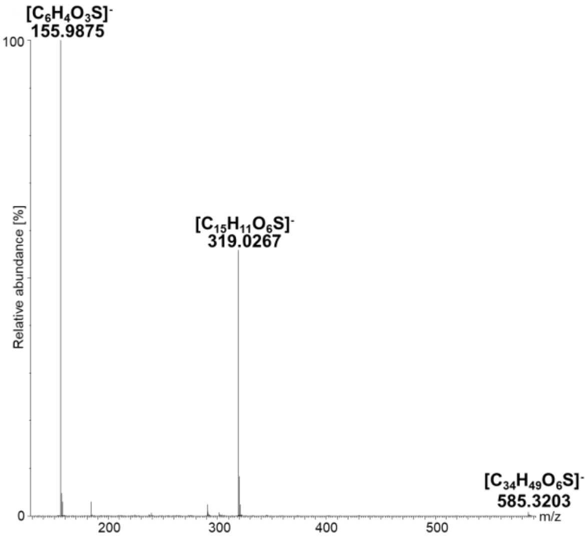

(H) γ-tocopherol

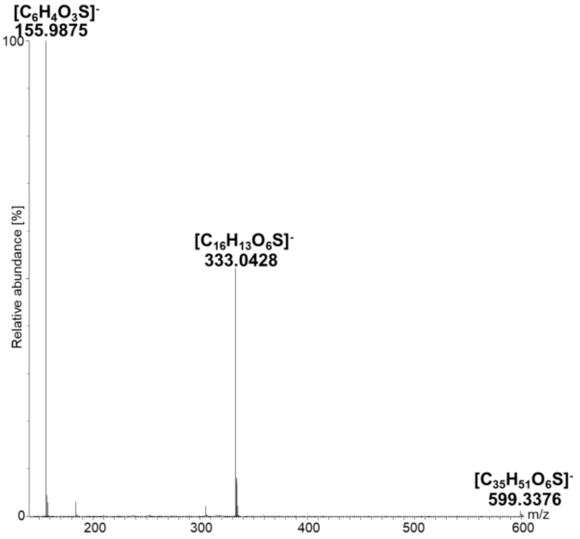

Figure S3: Effect of molar ratio (*v/v*) of pyridine and 3-(chlorosulfonyl)benzoic acid (Cl-SBA) on the MS signal response of studied lipid derivatives: **(A)** MG 18:1 D7 monosubstituted (1x), **(B)** MG 19:1 monosubstituted (1x), **(C)** MG 18:1 D7 disubstituted (2x), **(D)** MG 19:1 disubstituted (2x), **(E)** DG 28:0, **(F)** DG 36:2 D5, **(G)** DG 33:1 D7, **(H)** cholesterol D7, **(I)** desmosterol D6, **(J)** sitosterol D7, **(K)** α-tocopherol D6. Data are presented as the mean value ± standard deviation from three independent experiments.

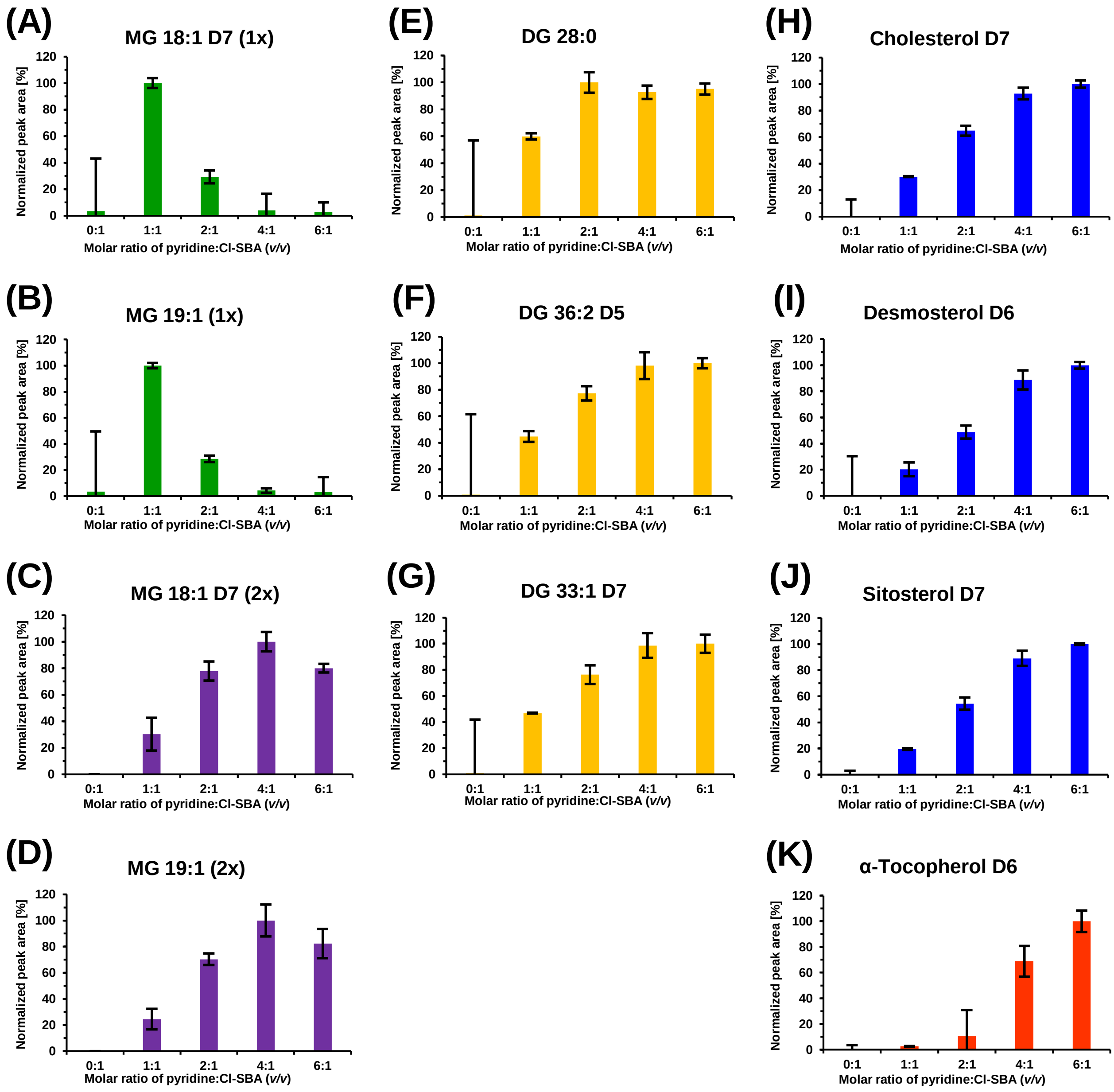

Figure S4: Effect of concentration of derivatization agent in acetonitrile (mg/mL) on the MS signal response of studied lipid derivatives: **(A)** MG 18:1 D7 monosubstituted (1x), **(B)** MG 19:1 monosubstituted (1x), **(C)** MG 18:1 D7 disubstituted (2x), **(D)** MG 19:1 disubstituted (2x), **(E)** DG 28:0, **(F)** DG 36:2 D5, **(G)** DG 33:1 D7, **(H)** cholesterol D7, **(I)** desmosterol D6, **(J)** sitosterol D7, **(K)** α-tocopherol D6. Data are presented as the mean value ± standard deviation from three independent experiments.

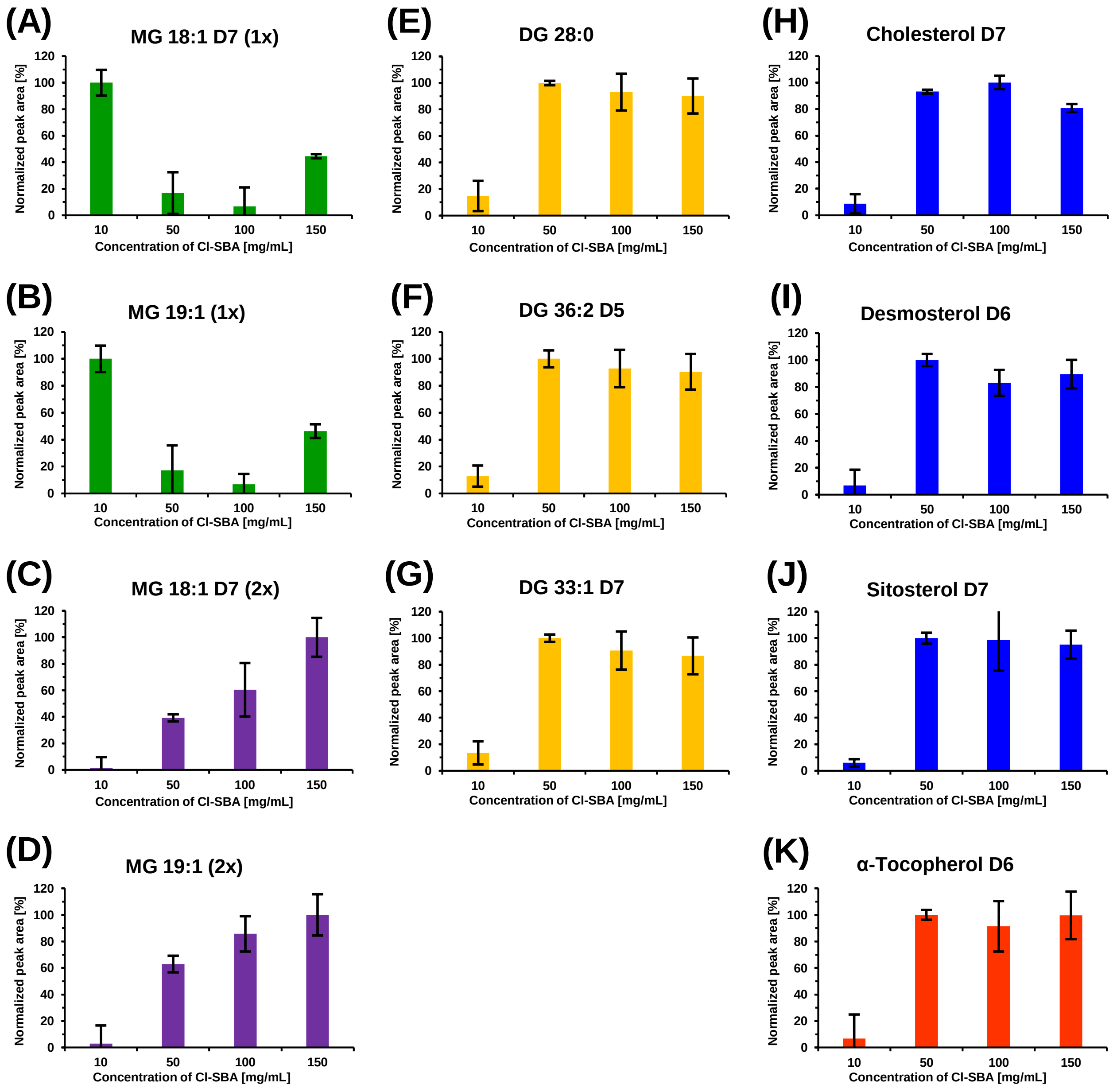

Figure S5: Effect of reaction temperature (°C) on the MS signal response of studied lipid derivatives: **(A)** MG 18:1 D7 monosubstituted (1x), **(B)** MG 19:1 monosubstituted (1x), **(C)** MG 18:1 D7 disubstituted (2x), **(D)** MG 19:1 disubstituted (2x), **(E)** DG 28:0, **(F)** DG 36:2 D5, **(G)** DG 33:1 D7, **(H)** cholesterol D7, **(I)** desmosterol D6, **(J)** sitosterol D7, **(K)** α-tocopherol D6. Data are presented as the mean value ± standard deviation from three independent experiments.

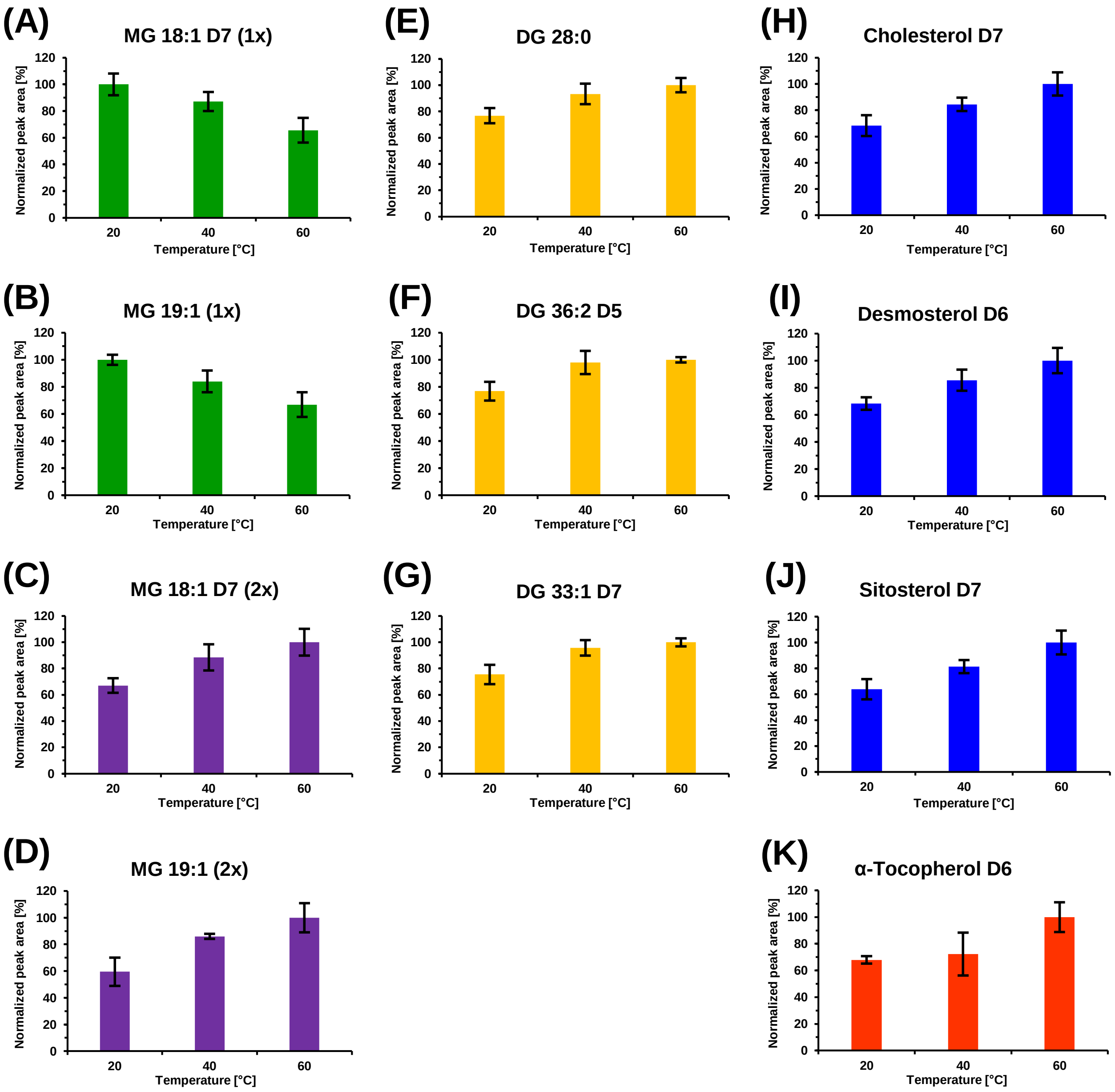

Figure S6: Effect of reaction time at 60 °C (min) on the MS signal response of studied lipid derivatives: **(A)** MG 18:1 D7 monosubstituted (1x), **(B)** MG 19:1 monosubstituted (1x), **(C)** MG 18:1 D7 disubstituted (2x), **(D)** MG 19:1 disubstituted (2x), **(E)** DG 28:0, **(F)** DG 36:2 D5, **(G)** DG 33:1 D7, **(H)** cholesterol D7, **(I)** desmosterol D6, **(J)** sitosterol D7, **(K)** α-tocopherol D6. Data are presented as the mean value ± standard deviation from three independent experiments.

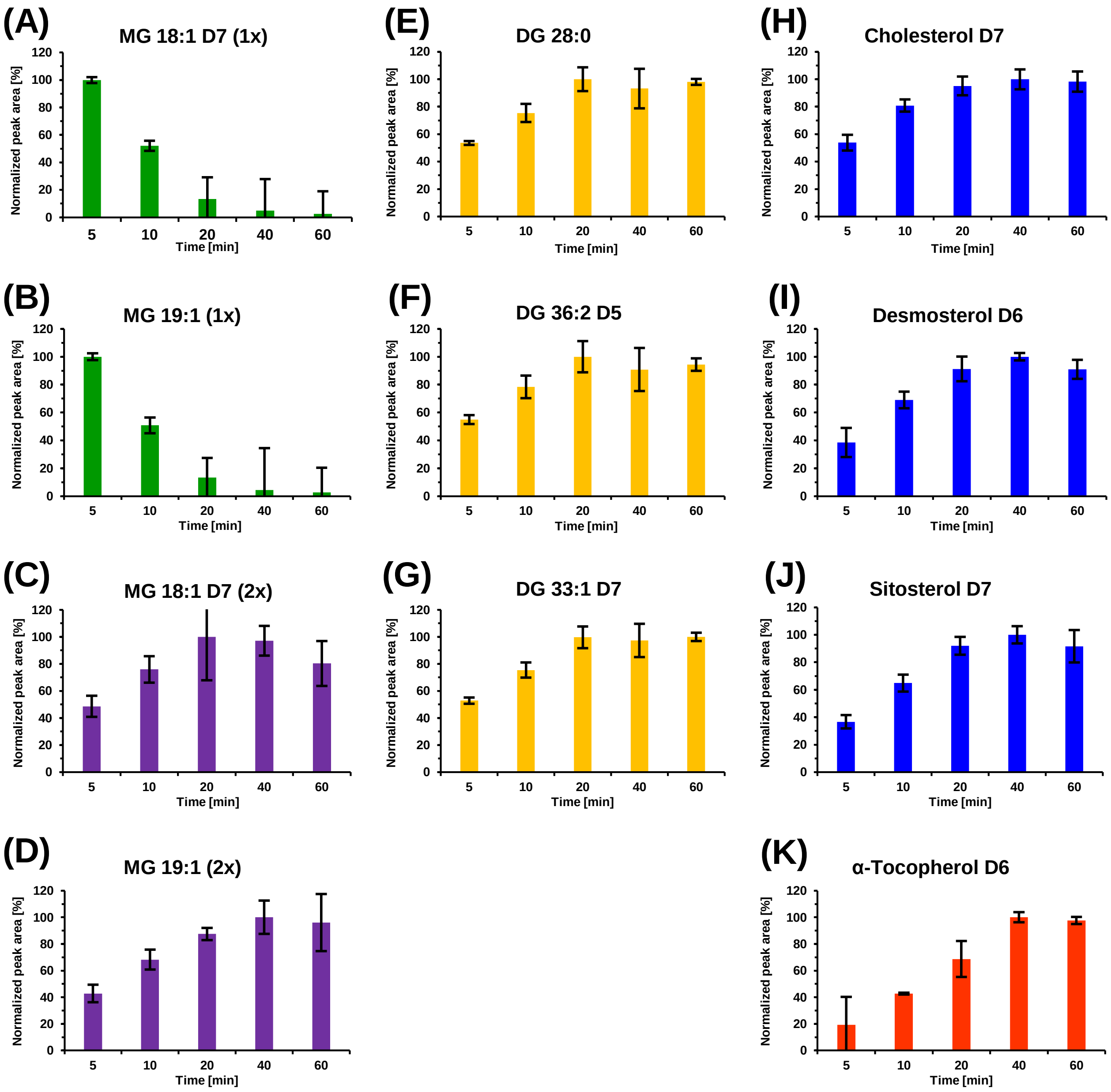

Figure S7: Optimization of composition of aqua phase for Folch extraction. Comparison of basic (250 mM of ammonium carbonate), neutral (pure water), and acidic (0.1% of formic acid) environment: **(A)** MG 18:1 D7 monosubstituted (1x), **(B)** MG 19:1 monosubstituted (1x), **(C)** MG 18:1 D7 disubstituted (2x), **(D)** MG 19:1 disubstituted (2x), **(E)** DG 28:0, **(F)** DG 36:2 D5, **(G)** DG 33:1 D7, **(H)** cholesterol D7, **(I)** desmosterol D6, **(J)** sitosterol D7, **(K)** α-tocopherol D6. Data are presented as the mean value ± standard deviation from three independent experiments.

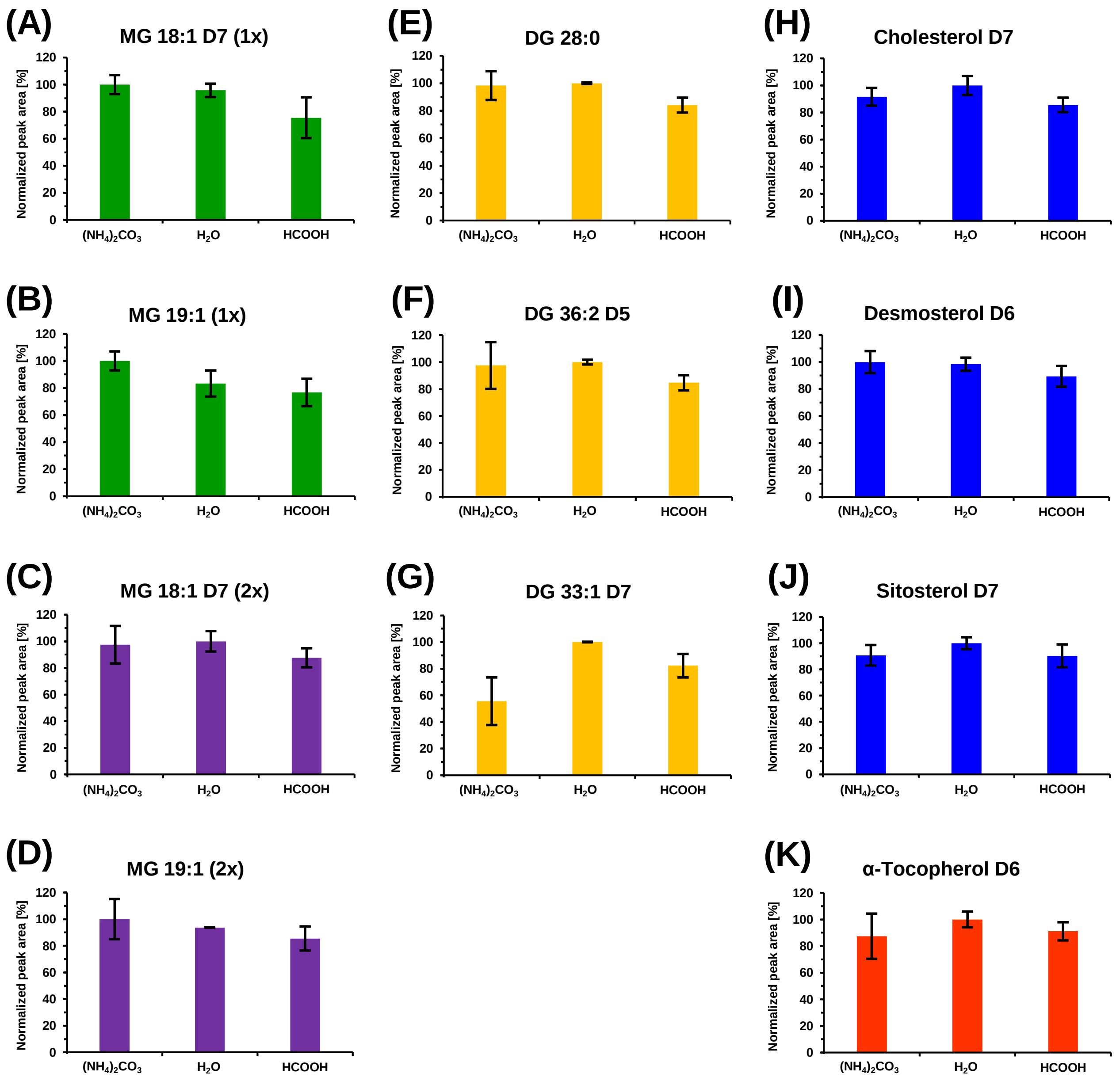

Figure S8: Short-term stability test investigated stability of derivates within 10 hours in an autosampler (4 °C): **(A)** MG 18:1 D7 monosubstituted (1x), **(B)** MG 19:1 monosubstituted (1x), **(C)** MG 18:1 D7 disubstituted (2x), **(D)** MG 19:1 disubstituted (2x), **(E)** DG 28:0, **(F)** DG 36:2 D5, **(G)** DG 33:1 D7, **(H)** cholesterol D7, **(I)** desmosterol D6, **(J)** sitosterol D7, and **(K)** α-tocopherol D6. Data are presented as the mean value ± standard deviation from three independent experiments.

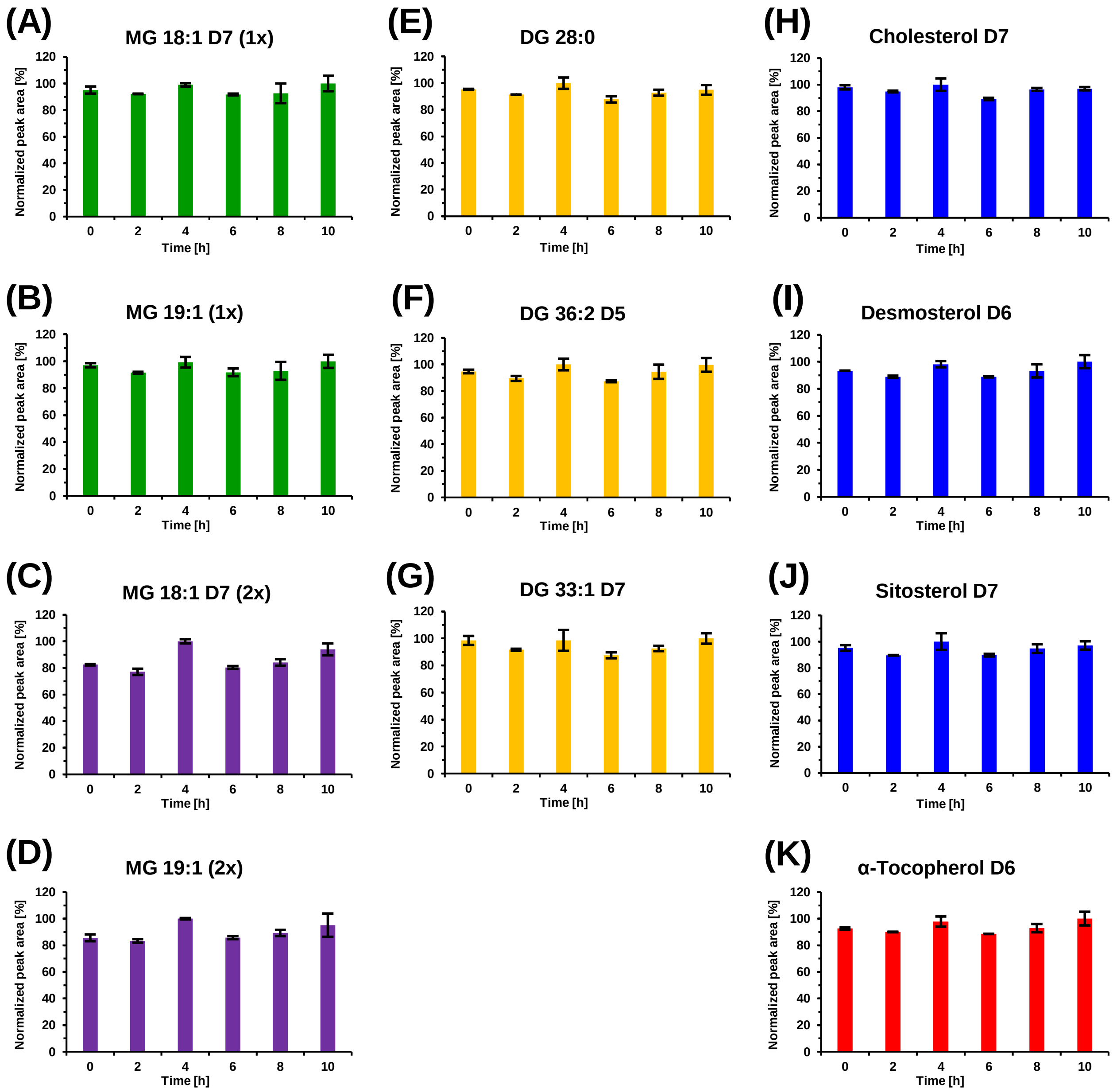

Figure S9: Long-term stability test investigated stability (store at -80°C) of derivates during 5 days: **(A)** MG 18:1 D7 monosubstituted (1x), **(B)** MG 19:1 monosubstituted (1x), **(C)** MG 18:1 D7 disubstituted (2x), **(D)** MG 19:1 disubstituted (2x), **(E)** DG 28:0, **(F)** DG 36:2 D5, **(G)** DG 33:1 D7, **(H)** cholesterol D7, **(I)** desmosterol D6, **(J)** sitosterol D7, and **(K)** α-tocopherol D6. Data are presented as the mean value ± standard deviation from three independent experiments.

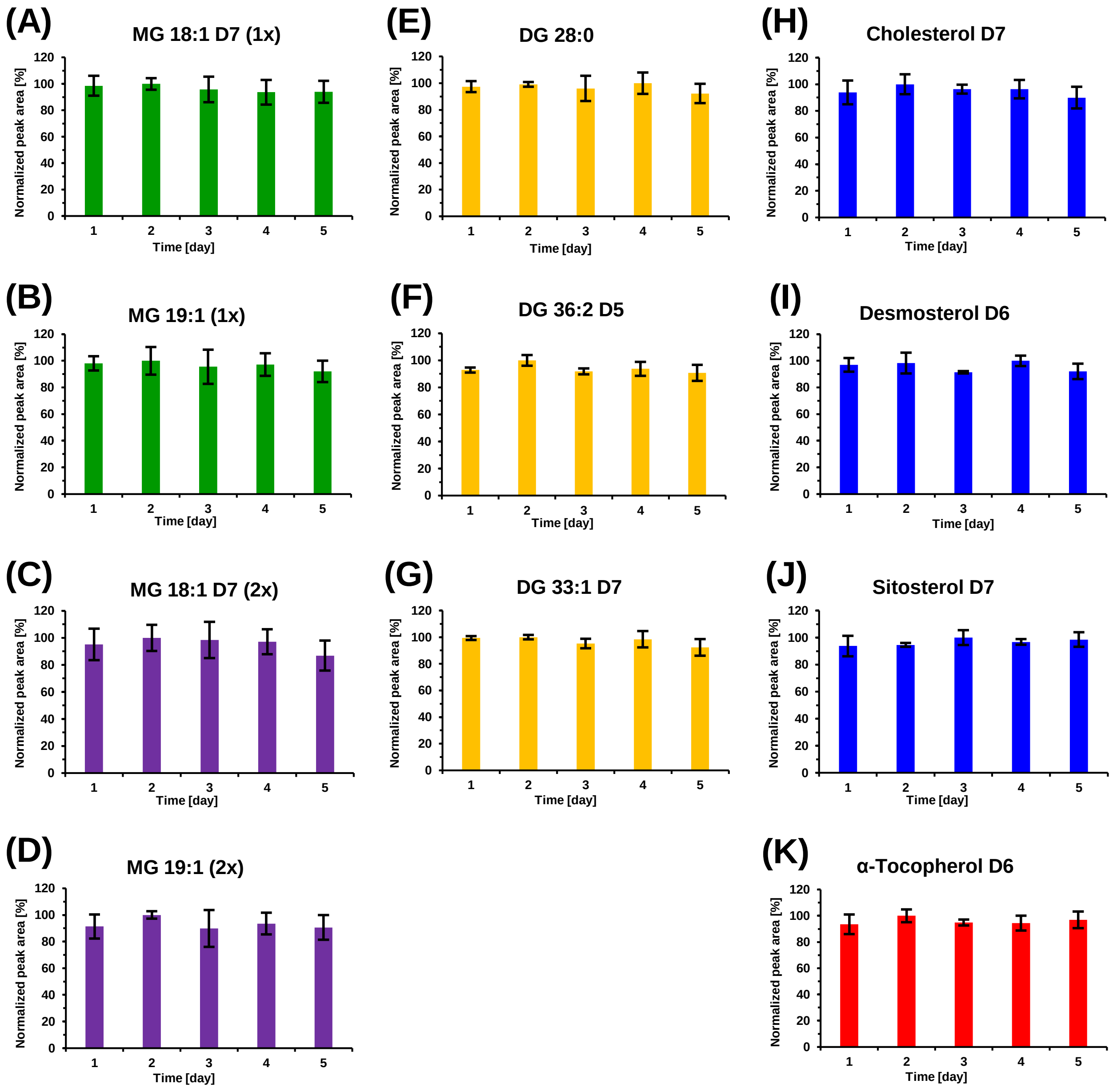

Figure S10: Optimization of mass spectrometry parameters using standard mix: **(A)** drying temperature (°C), **(B)** nebulizer gas (GS1) pressure (psi), **(C)** heating gas (GS2) pressure (psi), **(D)** curtain gas (CUR) pressure (psi). Data are presented as the mean value ± standard deviation from three independent experiments. Other derivatized lipid standards from the same lipid class follow the same trend.

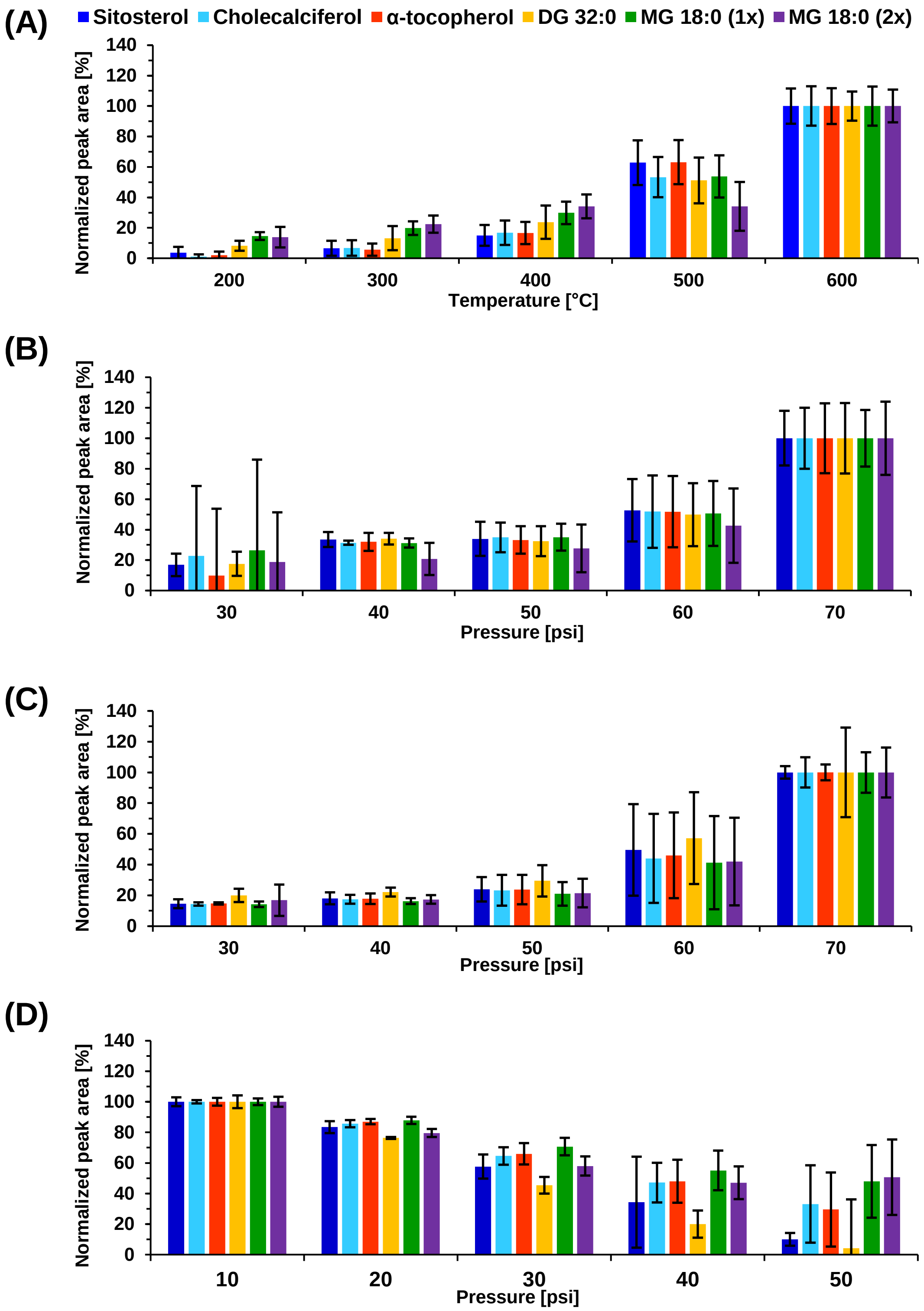

Figure S11: Extracted ion chromatograms of the derivatized standard of **(A)** monosubstituted monoacylglycerol (MG 1x), **(B)** disubstituted monoacylglycerol (MG 2x), **(C)** sterols and prenols, and **(D)** diacylglycerols.

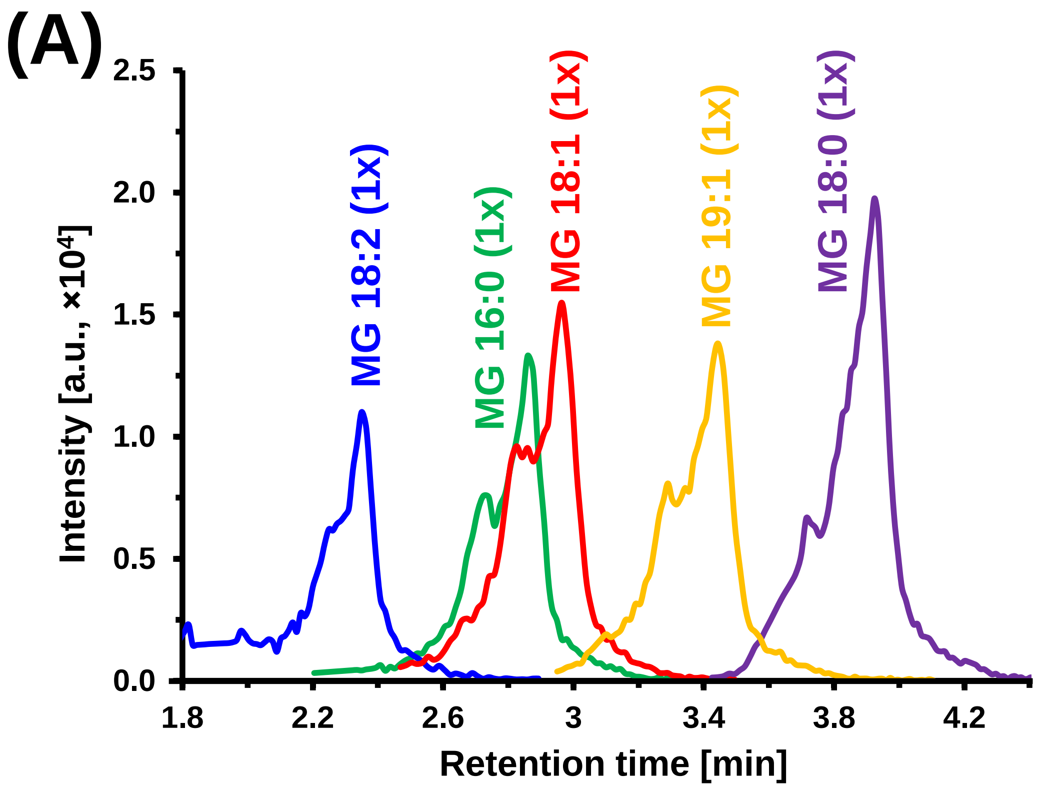

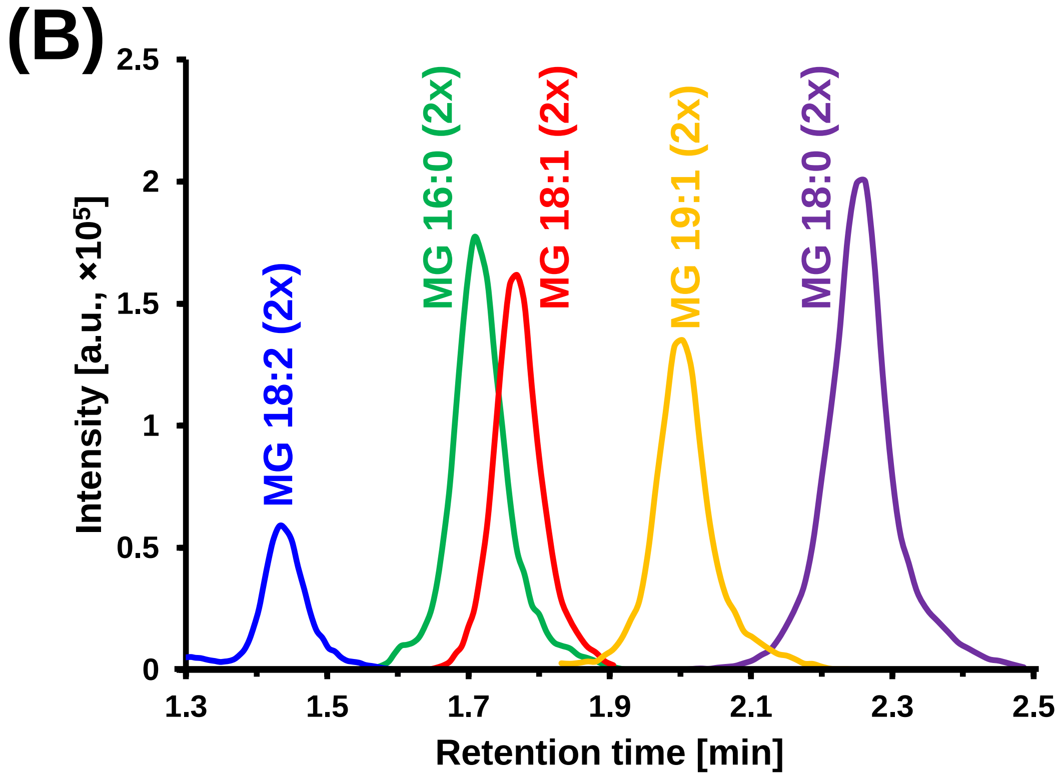

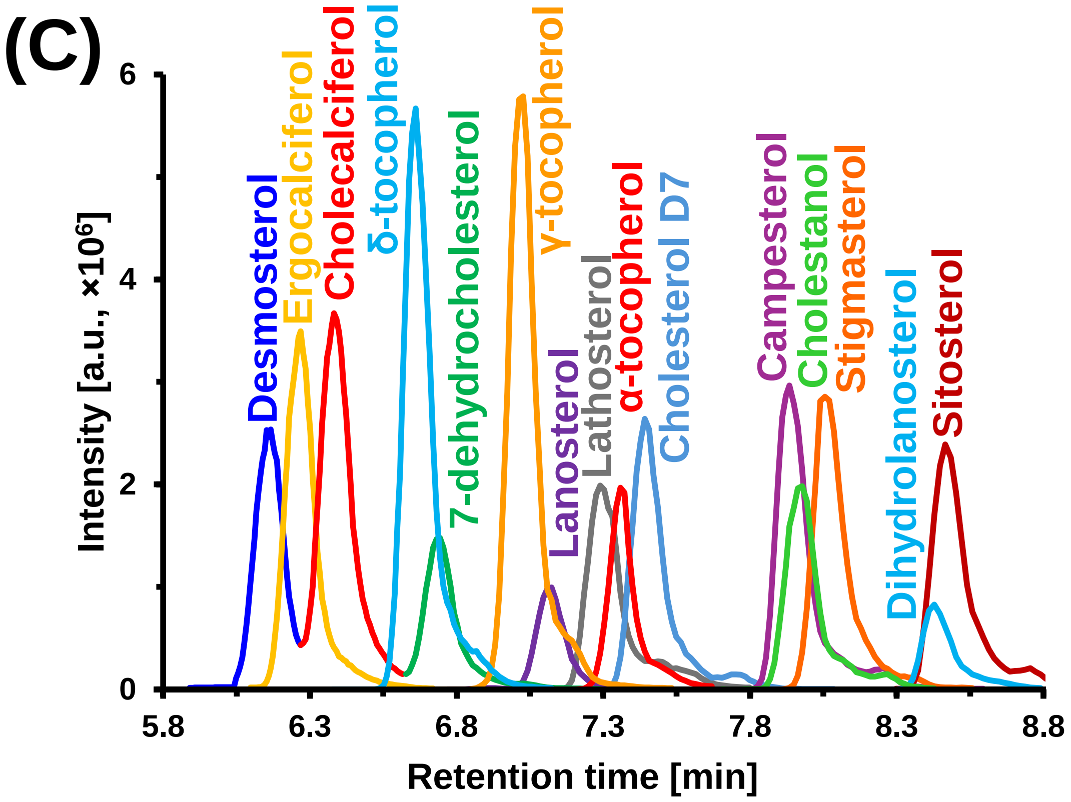
(C)
Intensity [a.u., ×10^6]
Retention time [min]
Desmosterol
Ergocalciferol
Cholecalciferol
δ-tocopherol
7-dehydrocholesterol
γ-tocopherol
Lanosterol
Lathosterol
α-tocopherol
Cholesterol D7
Campesterol
Cholestanol
Stigmasterol
Dihydrolanosterol
Sitosterol

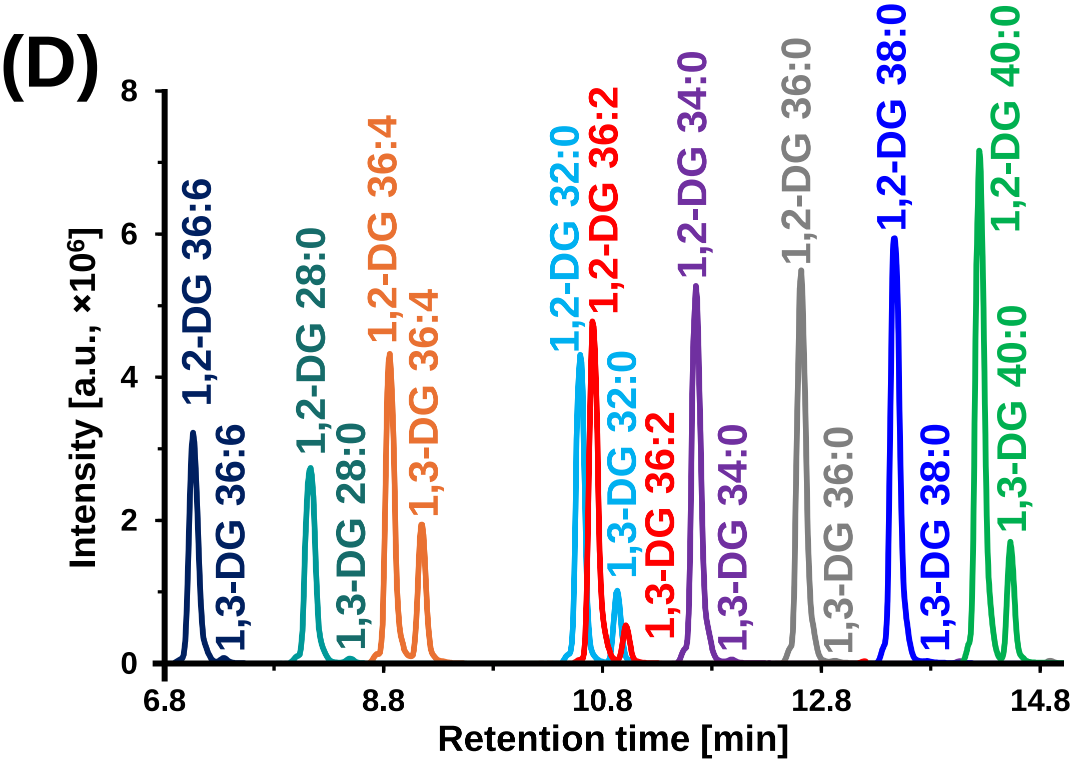
(D)
Intensity [a.u., ×10^6]
Retention time [min]
1,2-DG 36:6
1,3-DG 36:6
1,2-DG 28:0
1,3-DG 28:0
1,2-DG 36:4
1,3-DG 36:4
1,2-DG 32:0
1,2-DG 36:2
1,3-DG 32:0
1,3-DG 36:2
1,2-DG 34:0
1,3-DG 34:0
1,2-DG 36:0
1,3-DG 36:0
1,2-DG 38:0
1,3-DG 38:0
1,2-DG 40:0
1,3-DG 40:0

Figure S12: Extracted ion chromatograms of the derivatized isomeric sterols: **(A)** Desmosterol/Cholecalciferol/7-dehydrocholesterol and **(B)** Lathosterol/Cholesterol D7.

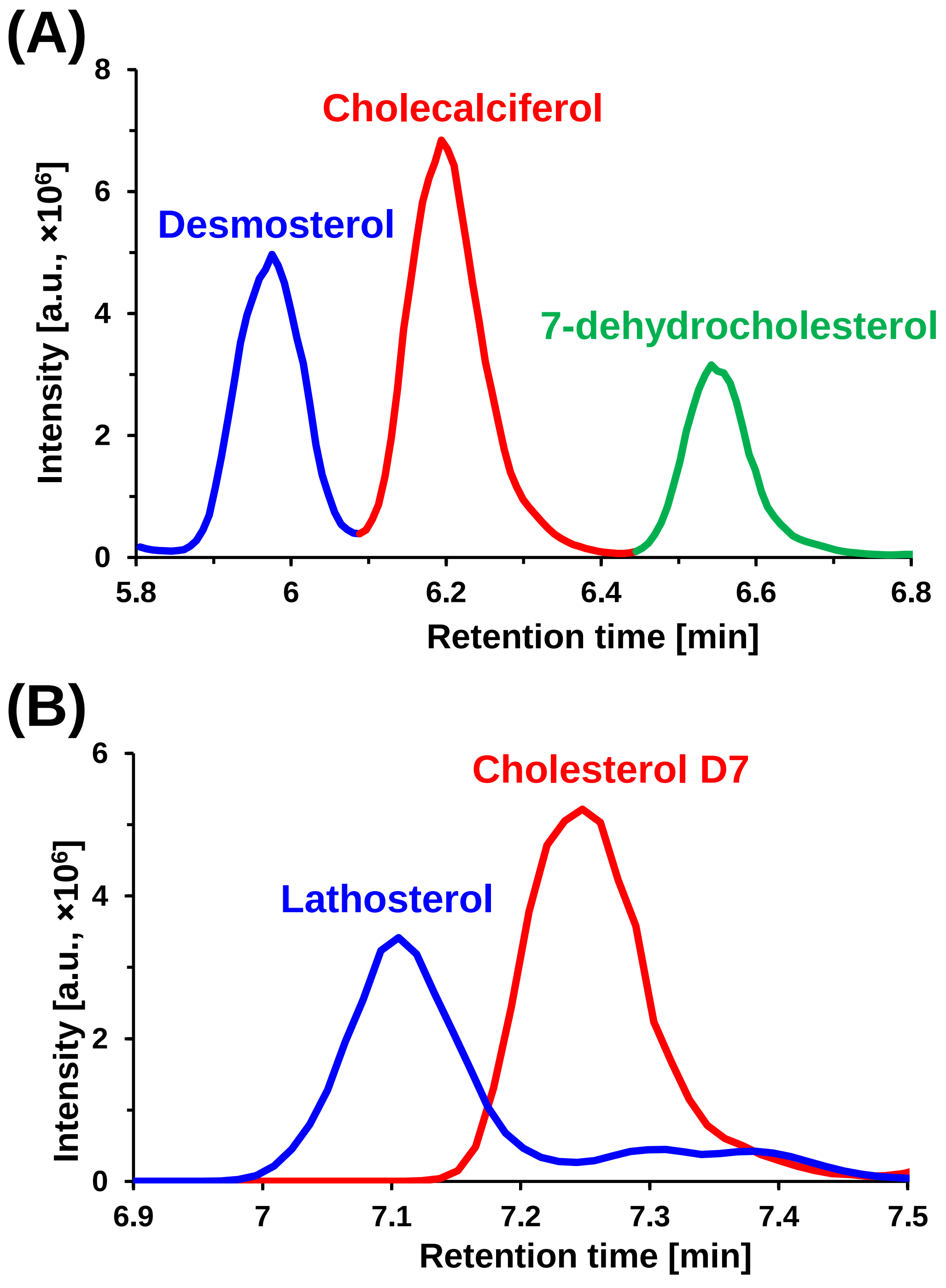

Figure S13: Repeatability of derivatization method investigated by spiked human plasma: **(A)** MG 18:1 D7 disubstituted (2x), **(B)** MG 19:1 disubstituted (2x), **(C)** α-tocopherol D6, **(D)** DG 28:0, **(E)** DG 33:1 D7, **(F)** DG 36:2 D5, **(G)** cholesterol D7, **(H)** desmosterol D6, and **(I)** sitosterol D7. Data present ten independent experiments, where the gray line represents median of values and orange line median ± 2 standard deviations.

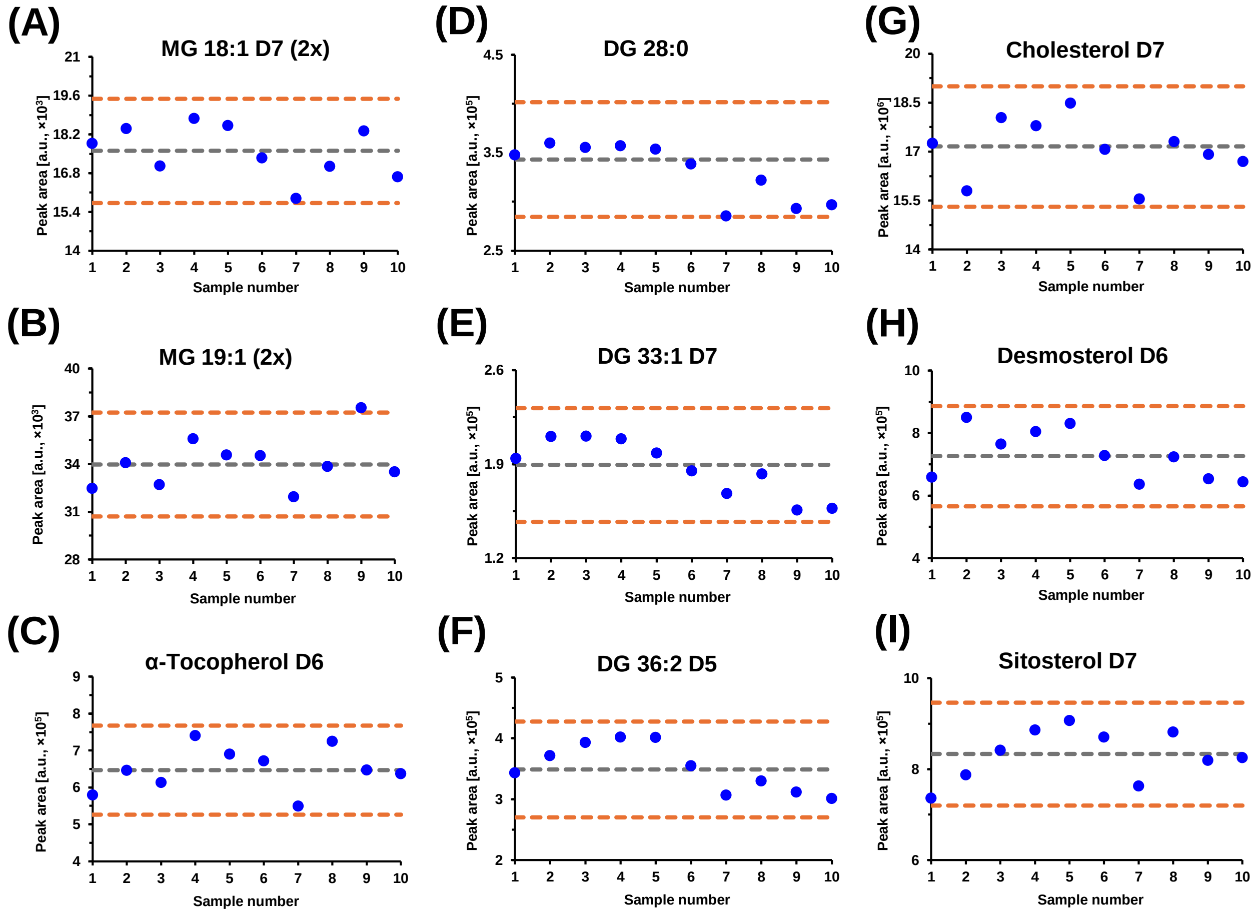

Figure S14: Calibration curves of derivatized internal standards in spiked human plasma: **(A)** MG 18:1 D7 disubstituted, **(B)** MG 19:1 disubstituted, **(C)** DG 28:0, **(D)** DG 33:1 D7, **(E)** DG 36:2 D5, **(F)** cholesterol D7, **(G)** desmosterol D6, **(H)** sitosterol D7, and **(I**) α-tocopherol D6. Data present the mean value of three independent experiments.

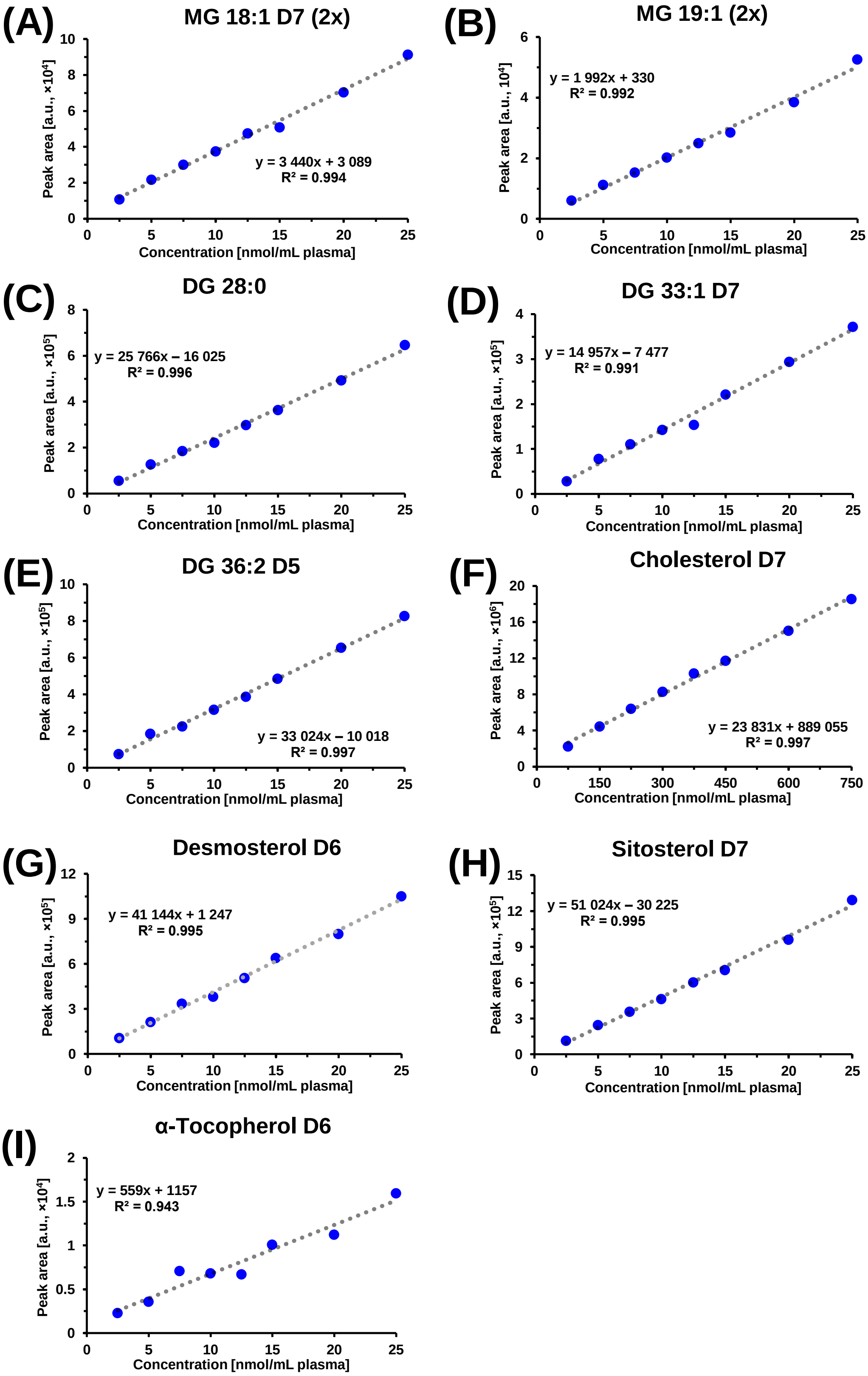

Figure S15: Graphical visualization of dependencies of the retention time on the carbon number, where X represents the carbon number and number of double bond(s) of individual lipids is written behind colon.

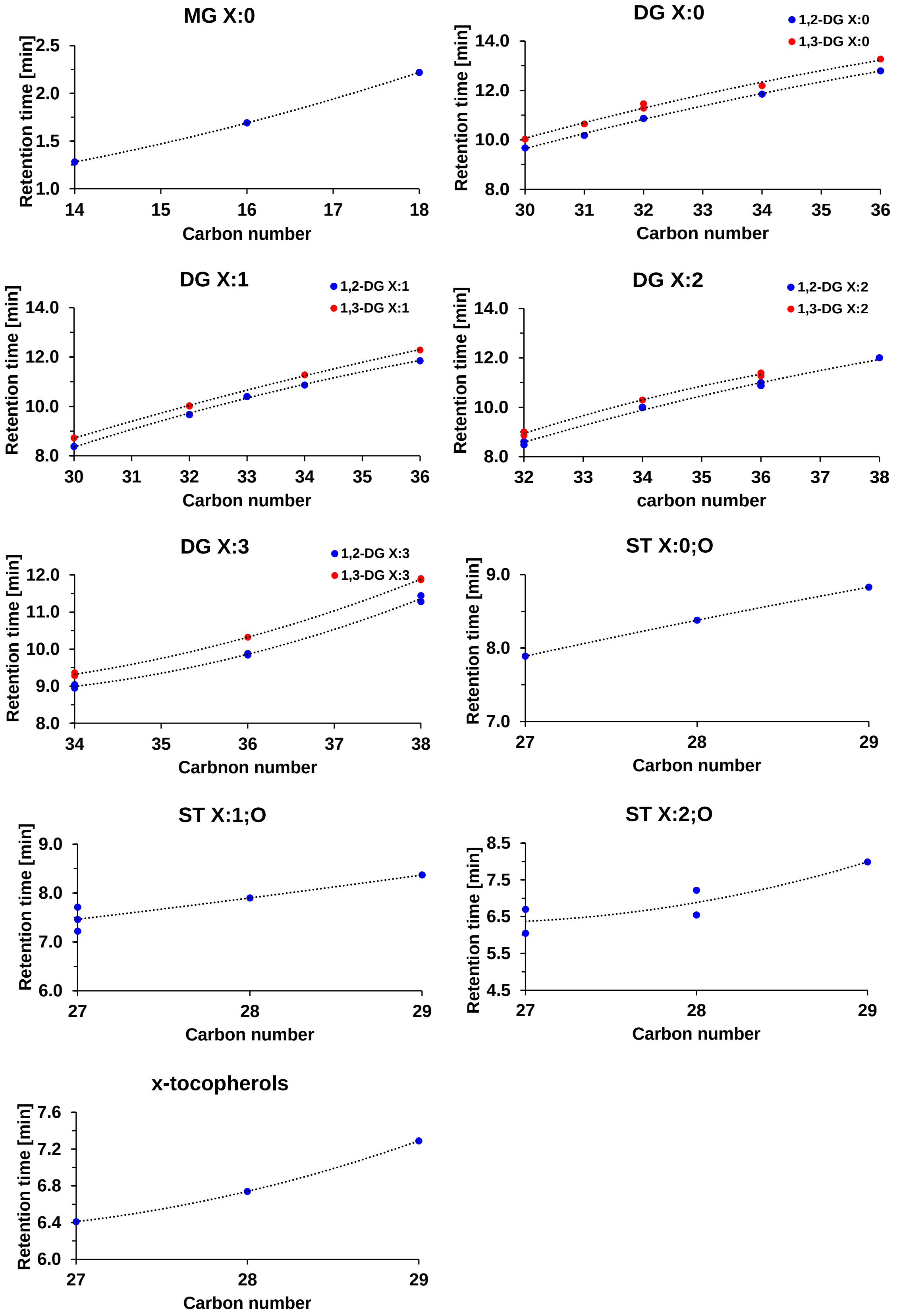

Figure S16: Graphical visualization of dependencies of the retention time on number of double bond(s), where Y represents the number of double bond(s) and the number of carbons of individual lipids is written before colon.

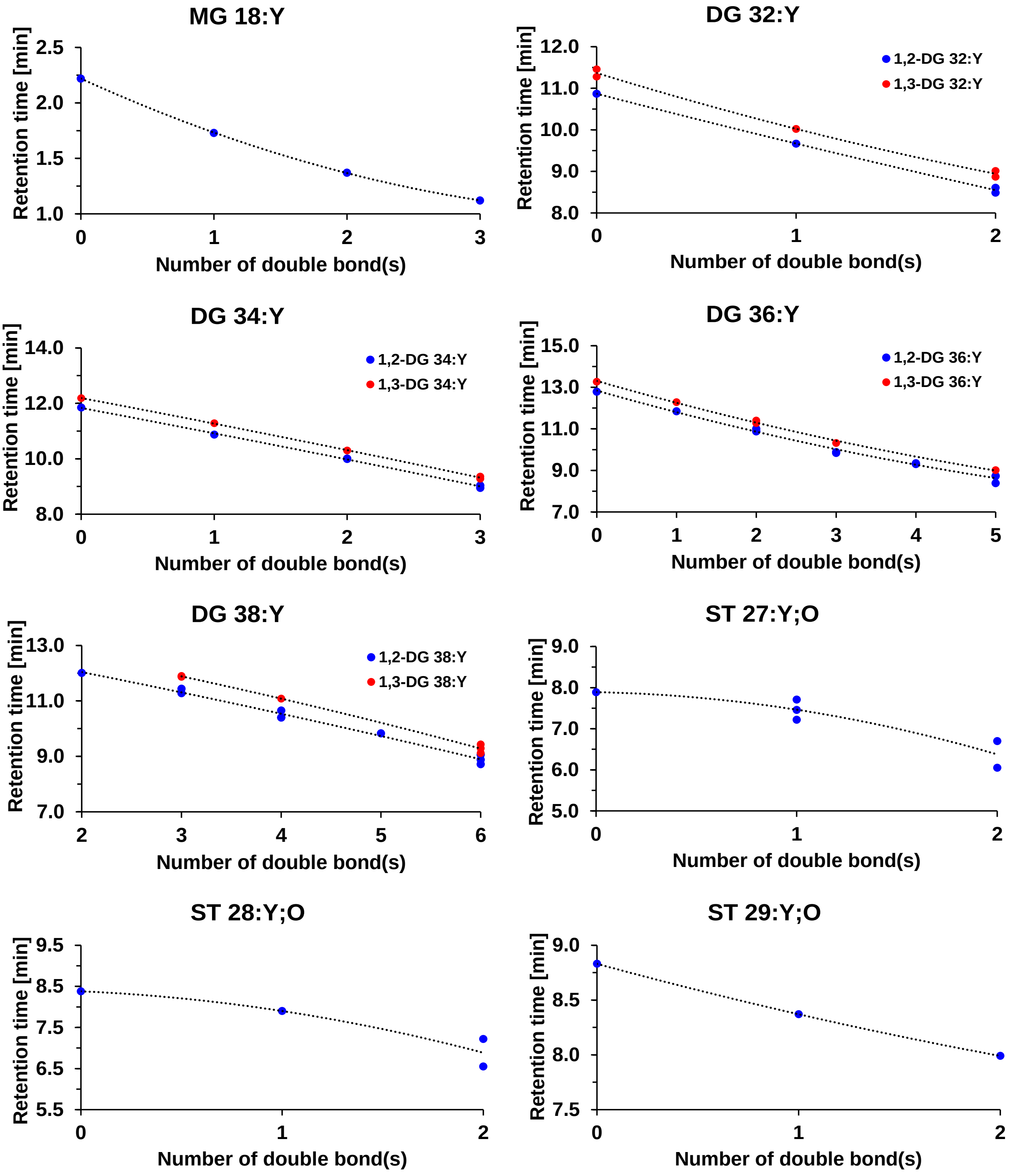

Figure S17: Chromatograms of derivatized plasma sample visualized **(A)** real chromatogram, **(B)** decreasing of cholesterol intensity 100 times, and **(C)** detail separation of ST 27:1;0 isomers (lathosterol/cholesterol/ST 27:1 (c)) and ST 27:0;O (cholestanol).

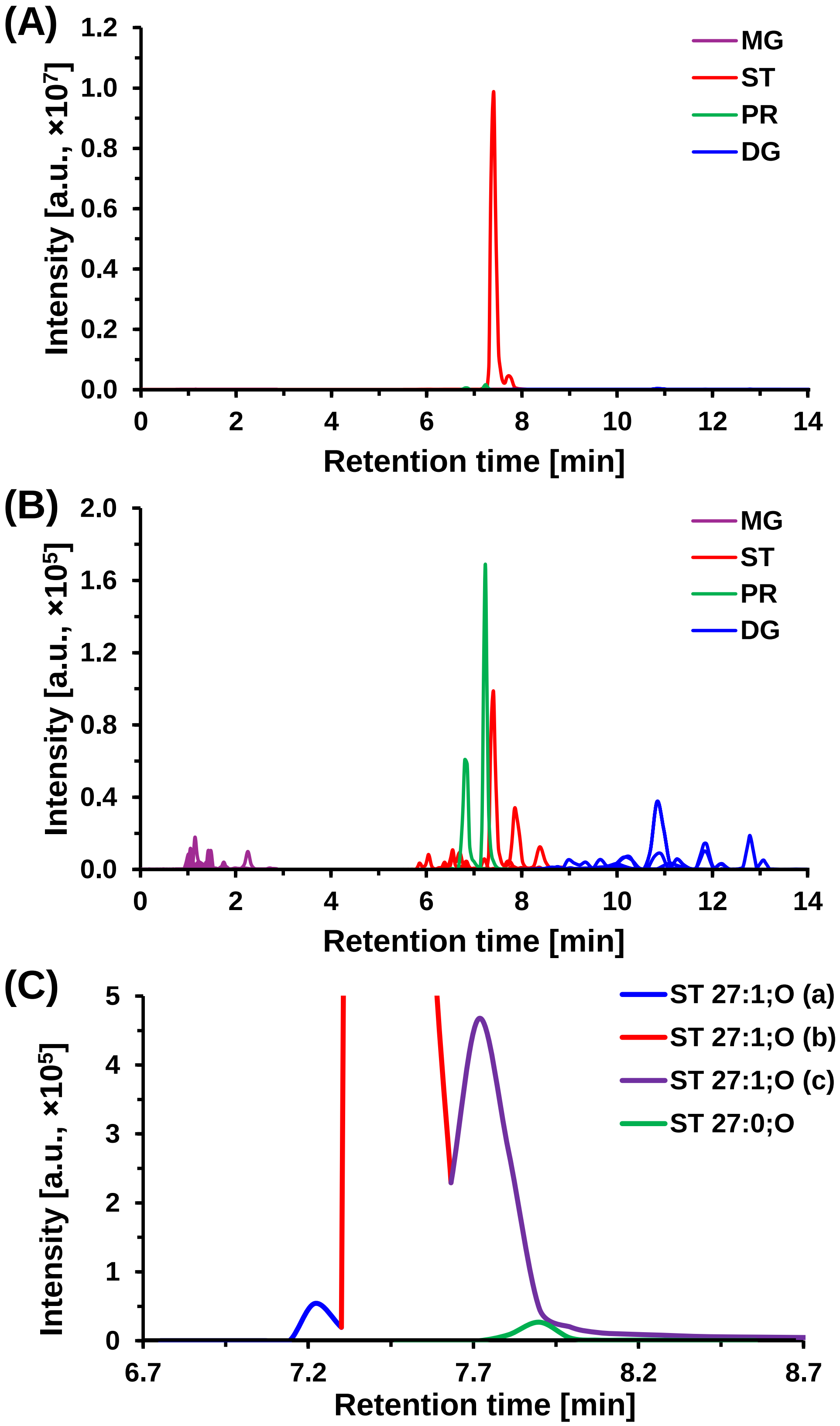

Supplement: Supplementary file 1 — ac4c06496_si_001.pdf [file ac4c06496_si_001.pdf]
